# Biofilm formation and role of other pathogenic factors in the virulence of *Staphylococcus epidermidis* clinical isolates

**DOI:** 10.3389/fcimb.2025.1630341

**Published:** 2025-08-06

**Authors:** María Coronada Fernández-Calderón, Irene Fernández-Babiano, María Luisa Navarro-Pérez, Carmen Pazos-Pacheco, Antonia Calvo-Cano

**Affiliations:** ^1^ Area of Microbiology, Department of Biomedical Sciences, Faculty of Medicine and Health Sciences, University of Extremadura, Badajoz, Spain; ^2^ Bioengineering Unit. University Institute of Biosanitary Research of Extremadura (INUBE), Badajoz, Spain; ^3^ Centre for Biomedical Research in Network, Bioengineering, Biomaterials and Nanomedicine (CIBER-BBN), Badajoz, Spain; ^4^ Clinical Microbiology Department, San Pedro de Alcántara Hospital, University Hospital Complex of Cáceres, Cáceres, Spain; ^5^ Area of Medicine, Department of Biomedical Sciences, Faculty of Medicine and Health Sciences, University of Extremadura, Badajoz, Spain

**Keywords:** *Staphylococcus epidermidis*, pathogenicity factors, adhesion, biofilm, hidrophobicity, medical-device infections, multidrug resistance

## Abstract

Medical device-associated infections represent a significant healthcare challenge, as sterilization of the biomaterial often necessitates device removal. The most frequently isolated microorganism in these infections is *Staphylococcus epidermidis*, a skin commensal capable of causing a wide range of nosocomial infections. The primary virulence factor of *S. epidermidis* is biofilm formation, which decreases antibiotic efficacy and host immune response. However, additional factors play crucial roles in infection establishment. Understanding the interplay between virulence factors is essential to developing preventive strategies that inhibit microbial adhesion and biofilm development. In this study, we analyzed the presence of genes associated with adhesion and biofilm formation (*ica*-dependent and *ica*-independent pathways) in 25 clinical isolates of *S. epidermidis* and four control strains: ATCC 12228, ATCC 35983, ATCC 35984, and the HAM 892 mutant. Resistance profile was determined, and biofilm quantification and composition of matrix was performed using multiple methodologies. Additionally, parameters associated with initial adherence as cell surface hydrophobicity (CSH) were investigated. A strong correlation was observed among different methods for measuring biofilm formation and matrix composition. The 14 *icaADBC*+ isolates exhibited higher prevalence of the *aap*, *bhp*, *mecA*, and *IS256* genes, with polysaccharide intercellular adhesin (PIA) identified as the main matrix component. In contrast, *icaADBC*− biofilm-producing strains formed biofilms rich in other polysaccharides and proteins. The 15 non-biofilm-producing isolates showed significantly higher hydrophobicity levels, suggesting that this factor plays a critical role in initial adhesion and colonization. This study highlights the diverse mechanisms underlying biofilm formation in *S. epidermidis* and identifies hydrophobicity as a potential pathogenicity factor contributing to its virulence.

## 1 Introduction

Biomaterial-associated infections represent one of the most serious clinical complications, affecting a wide range of medical and surgical devices, and exerting a significant impact in terms of morbidity, mortality, and healthcare costs (Campoccia et al., 2006). The pathogenesis of these infections depends on the interplay of four key factors: the microorganism, host defense mechanisms, the biomaterial, and antimicrobial agents (Kleinschmidt et al., 2015). Effective treatment usually requires removal of the infected device, sometimes in combination with prolonged antimicrobial therapy (Zimmerli and Moser, 2012). The microorganisms most commonly implicated in these infections are coagulase-negative staphylococci (CoNS), particularly *Staphylococcus epidermidis* (Schilcher and Horswill, 2020). Device-related infections typically progress through several stages: initial reversible adhesion, an accumulative phase involving irreversible junctions and formation of bacterial aggregates, and finally, the dispersion of cells that migrate to establish new sites of infection. Each stage is driven by distinct pathogenicity factors. These biofilm-promoting elements are integrated into regulatory networks that play a crucial role in the development and persistence of staphylococcal infections (Schilcher and Horswill, 2020).

In the early stage of infection, bacterial adhesion to abiotic surfaces is critical and depends on bacterial traits like cell surface hydrophobicity (CSH) (Schilcher and Horswill, 2020) and ATP dependant proteases like *Clp* peptidase (Wang et al., 2007), as well as substrate properties (Zheng et al., 2021). Upon contact with the biomaterial, bacteria proliferate and form monolayers using adhesins and capsular polysaccharides (von Eiff et al., 2001). Proteins encoded by *altE* and *Bhp* (a biofilm-associated protein [*Bap*] homologue) facilitate adhesion through hydrophobic interactions, with *AltE* also promoting extracellular DNA release via autolysis (Foster, 2020; Zhang et al., 2024). In *S. epidermidis*, hydrophobicity—linked to wall components like lipoteichoic acids (LTA) and proteins—is a key factor in initial adhesion (Gottenbos et al., 2000; Schilcher and Horswill, 2020).

During the accumulative phase, bacterial cells proliferate and produce an extracellular polymeric matrix that anchors them to each other and to the surface (Sutherland, 2001). In staphylococci, this matrix is primarily composed of a β-1,6-N-acetylglucosamine structure (PNAG) (Gotz, 2002). Initially termed polysaccharide intracellular adhesin (PIA) (Götz and Peters, 2000), this polymer is the main component responsible for intercellular adhesion in *S. epidermidis* biofilms (Mack et al., 1996). PIA/PNAG is crucial for biofilm formation and accumulation (Mack et al., 1996; Maira-Litran et al., 2004), protects the pathogen from innate immune responses (Vuong et al., 2004), and contributes to the hemagglutinin activity of *S. epidermidis.* This latter function promotes erythrocyte binding and aggregation, enhancing protection against host defenses (Gotz, 2002; Otto, 2009).

PIA/PNAG synthesis is mediated by enzymes encoded by the intercellular adhesin cluster (*ica*) operon. This locus includes five genes (*icaADBC* and the negative regulator *ica*R) and is present in most *S. epidermidis* strains. The slime-positive phenotype has been linked to increased virulence and persistence in animal models (Vuong et al., 2004). However, some slime-negative strains can also cause infections if they express alternative virulence factors (Rohde et al., 2010).

In addition to PIA, alternative mechanisms of cell accumulation and biofilm formation independent of the *ica* locus have been identified. These include surface proteins such as *Aap* (*S. epidermidis* accumulation-associated protein), *Embp* (extracellular matrix-binding protein), and *Bhp*. *Embp* is a large fibronectin-binding protein that facilitates both intercellular adhesion and extracellular matrix binding without relying on polysaccharides (Conlon et al., 2014). *Bhp* can also promote cell accumulation in certain strains (Foster, 2020). The *aap* gene encodes a repeat-rich surface protein whose expression is regulated by environmental conditions and the *agr* quorum sensing system (Schilcher and Horswill, 2020). Although detection of *icaADBC* has been proposed as a marker to distinguish invasive from commensal strains in clinical isolates, it is not definitive. Other markers include the *mecA* gene, which confers methicillin resistance, and factors involved in foreign body colonization such as *AtlE*, *Fbe*, and *Aap*. However, some studies report no significant differences in these markers between invasive and commensal strains (Rohde et al., 2005).

Genetic elements such as insertion sequences (IS) may contribute to phenotypic variation in *S. epidermidis*. Among them, *IS256—*a mobile genetic element presents in multiple chromosomal copies—has been associated with virulence, likely through the expression of a transposase (Kozitskaya et al., 2004). *IS256* can influence *ica* gene expression, potentially leading to a biofilm-negative phenotype (Kalantar-Neyestanaki et al., 2023). It is hypothesized that *IS256* affects the expression of various pathogenicity-related genes, and its multiple copies may enhance genomic plasticity and environmental adaptability (Gu et al., 2005). Consequently, *IS256* is considered a virulence-associated marker in invasive strains (Gu et al., 2005; Kozitskaya et al., 2004). The co-detection of *ica*A/D and *IS256* has been strongly correlated with robust biofilm formation and treatment failure in coagulase-negative staphylococcal infections (Diemond-Hernández et al., 2010; Chopjitt et al., 2025). Therefore, this study aimed to genetically characterize the selected *S. epidermidis* strains and analyze them for the presence of the following genes: *AtlE*, *Fbe*, and *ClpP* (involved in initial surface attachment); *icaADBC*, *IS256* and *IS257* (associated with *ica*-dependent cell accumulation); and *aap*, *bhp*, and *Embp* (involved in *ica*-independent aggregation, considering that *aap* and *Embp* encode multifunctional proteins also playing key roles in surface colonization) (Schilcher and Horswill, 2020). The *mecA* gene was also included as a biomarker of resistance and pathogenicity. In addition, the study assessed surface characteristics such as cell surface hydrophobicity, biofilm forming capacity, and biofilm composition. Finally, it aimed to identify potential differences in the initial adhesion phase between bare artificial surfaces and those pre-coated with host-derived proteins (fibronectin).

This article explores the hypothesis that understanding the interplay between various *S. epidermidis* pathogenicity factors may provide a deeper insight into the infection process and support the development of preventive strategies for the clinical use of biomaterials.

## 2 Materials and methods

### 2.1 Microorganisms and culture conditions

Twenty-five strains of *S. epidermidis* isolated from various clinical sources in the Microbiology Service of the University Hospital of Badajoz (Spain) were used. Four control strains were also used: three strains were obtained from the American Type Culture Collection (ATCC 12228; ATCC 35983; ATCC 35984), and the mutant HAM 892, which lacks the property of producing biofilm, by mutagenesis with acriflavine (Christensen et al., 1990), was from the Laboratory of Ultrastructure, Superiore Institute Sanita, Rome (courtesy of Dr. L. Baldassari). The biofilm-producing *S. epidermidis* strain, RP62A (ATCC 35984) (Christensen et al., 1982), and its mutant HAM 892 were used in all assays as positive and negative control, respectively. In addition, ATCC 35983, also used as positive control, was included to compare with a moderate biofilm-forming strain. The non-biofilm-producing *S. epidermidis* strain ATCC 12228 was also used as a negative control in all biofilm formation assays.

The identification of the strains was performed by conventional methods: Gram staining, morphological and culture characteristics, and production of catalase and coagulase. Finally, identification was confirmed using the API-Staph System Kit (bioMérieux, Marcy-L’Etoile, France).

From stock at −80°C in porous beads (Microbank, Pro-Labo Diagnostics), all strains were initially cultured in agar medium (Standard Methods Agar, APHA, Cultimed, Panreac Quimica S.A., Barcelona) and incubated at 37°C for 48 h. From these cultures, the bacteria were incubated in liquid culture medium, trypticase soy broth (TSB, BBL™ BD, Becton, Dickinson and Company, Sparks, NV, USA) at 37°C for 18–24 h.

### 2.2 Genetic characterization of *S. epidermidis* strains

General DNA manipulations were performed according to standard procedures. The gene amplification was carried out by PCR with specific primers designed from genomic sequences published in GenBank. PCR was performed with a thermocycler, MyCycler Personal Thermal Cycler (Bio-Rad Laboratories S.A., Madrid), in a final volume of 25 μl.

Mixtures for the amplification contained 25 pmol of each primer, 4 mM dNTPs mix, 10x buffer 50 mM MgCl2, and Taq polymerase (Biotools B&M Labs S.A., Madrid, Spain) used according to the manufacturer’s instructions. The PCR products sizes were analyzed by agarose gel electrophoresis at 1% (w/v) (Biotools B&M Labs S.A., Spain). Genes and conditions for each amplification are shown in **Supplementary Table S1**.

### 2.3 Antimicrobial susceptibility testing

Species identification and sensitivity to oxacillin as well as to other antibiotics were performed in a standardized manner in the laboratory using phenotypic methods like biochemical tests and automatic microdilution contained in the MicroScan PC 37 panels (Beckman Coulter).

The basis of all susceptibility testing is the minimum inhibitory concentration (MIC), and the categorizations of sensitive or resistant for each antibiotic are based on breakpoint for interpretation of these MICs of European Committee on Antimicrobial Susceptibility Testing Breakpoint (EUCAST) version 15.0, valid from 2025-01-01.

The antimicrobial susceptibility testing (AST) results were categorized as susceptible (S) or resistant (R) based on the MIC values.

Susceptibilities to 22 antibiotics of different families were tested: β-lactams (cefoxitin, cloxacillin, amoxicillin–clavulanate, penicillin), aminoglycosides (gentamicin, amikacin, tobramycin), fluorquinolones (ciprofloxacin and levofloxacin), macrolides (erythromycin), lincosamides (clindamycin), oxazolidiones (linezolid), sulfonamides (cotrimoxazole), ryfamicins (rifampicin), glycopeptides (vancomycin and teicoplanin), lipopetid (daptomycin), tetracyclines, and others such as fusidic acid, fosfomycin, nitrofurantoin, and mupirocin. Multidrug-resistant *S epidermidis* was defined by resistance to three or more families of antibiotics.

### 2.4 Characterization of *S. epidermidis* biofilm

#### 2.4.1 Quantitative and qualitative determination of biofilm production

Three methods were used to determine biofilm formation by *S. epidermidis* strains, which are shown in **Supplementary Figure S1A**: a semi-quantitative one and two qualitative methods. The semi-quantitative method was the spectrophotometric method microtiter plate test proposed by Stepanovic (Stepanovic et al., 2000), which in turn is based on the original method of Christensen (Christensen et al., 1985), but with some modifications. Those used as qualitative methods were the observation of slime production on tubes (Christensen et al., 1982) and the Congo red agar (CRA) assay (Freeman et al., 1989).

For biofilm production (quantitative determination), the inoculum was prepared from a TSB culture in exponential phase, which was initially adjusted to a transmittance of 82%, corresponding to 0.5 on the McFarland scale by means of a spectrophotometer (Helios Epsilon model, Thermo Spectronic, Waltham, MA, USA), and then diluted 1/100 to obtain an inoculum of approximately 10^6^ cfu/ml. This bacterial suspension was grown in sterile, 96-well, flat-bottomed polystyrene microtiter plates (Greiner bio-one, Frickenhausen, Germany). After 18–24 h of incubation, the contents of the wells were removed, and the wells were washed three times with 200 µl of 10 mM phosphate buffer (PBS) at pH 7.2 to remove all unattached bacteria. The remaining bacteria, which remained attached to the bottom of the wells, were fixed with 99% methanol (Panreac Quimica S.A., Barcelona) for 15 min and, after removing the methanol, were allowed to air dry.

The wells, once dry, were stained with 200 µl of crystal violet solution (VC, Gram-Hucker DC; Panreac, Barcelona, Spain) for 5 min. Excess dye was removed by successive washes with tap water. Again, the microtiter plates were allowed to air dry. Once dried, the OD of each well was measured at a wavelength of 492 nm using the automatic microplate reader. In order to resolubilize the dye attached to bacteria, 200 µl of 33% (v/v) glacial acetic acid (GAA, Panreac Quimica S.A., Barcelona) was added to each well and was left to act for 20 min. The amount of biofilm was determined by measuring the OD of each well at a wavelength of 492 nm, using an automatic microplate reader (ELx800, Bio-Tek Instruments, Inc, Winooski, VT, USA). Each assay was performed in triplicate and repeated on at least three different days.

For qualitative determinations, in the visualization technique, the strains were determined to be biofilm-forming when a visible film lined the wall and bottom of the glass tube. By Congo red agar technique, the agar plates were incubated at 37°C for 48 h until colonies were obtained. Black-pardusca colonies were considered as positive variants for biofilm production, while red-pink colonies were considered as negative.

#### 2.4.2 Detection of PIA/PNAG by hemagglutination test

Hemagglutination assays were performed according to Rupp laboratory (Rupp and Archer, 1992), with some modifications. *S. epidermidis* strains were incubated in TSB at 37°C for 18 h. The suspensions were sonicated in bath (J.P. Selecta S.A., Abrera, Barcelona) for 5 min, collected by centrifugation, and washed once with PBS containing 0.1% bovine albumin serum (BSA; Sigma, St. Louis, USA). Suspensions were adjusted to a transmittance of 31% in PBS with 0.1% BSA at a wavelength of 492 nm, equivalent to a McFarland standard 3, to obtain approximately 10^8^ cfu/ml. Sterile, 96-well, U-bottom microtiter plates (Greiner bio-one, Frickenhausen, UK) were used for assay. The bacterial suspension was initially diluted 1/2 in PBS, and successive serial dilutions were performed in the wells of the microplate. 50 μl of the sample and 50 μl of a 1% erythrocyte solution (red blood cells, human group B; Sigma, St. Louis, USA) in PBS with 0.1% BSA were added to each well. Finally, it was incubated at room temperature for 2 h. Positive assay was determined when a diffuse deposit was visualized at the bottom of the well. The assay was performed at least three times, in different days.

#### 2.4.3 Detection of PIA/PNAG by dot blot assay

PIA/PNAG in *S. epidermidis* biofilm was detected by dot blot method (Cramton et al., 1999). Bacteria were grown for 18 h in TSB, cell weight was determined, and the same number of cells from each of the cultures was resuspended in 50 µl of 0.5M EDTA (pH 8.0). The bacteria were incubated for 5 min at 100°C and centrifuged until a cell pellet was obtained. Supernatant (40 µl) was taken and incubated with 10 µl of proteinase K (20 mg/ml; Sigma-Aldrich Chemie GmbH P.O., Steinheim, Alemania) for 30 min at 37°C. Subsequently, 10 µl of Tris salt buffer (20 mM Tris-HCl, 150 mM NaCl [pH 7.4]) containing 0.01% bromophenol blue was added. 5 µl of this solution was deposited on a nitrocellulose filter using a Bio-Dot microfiltration apparatus (Bio-Rad Laboratories, Inc., Spain). Subsequently, the membrane was blocked overnight with 5% nonfat milk (AppliChem GmbH, Germany) in PBS with 0.1% Tween20. Finally, the membrane was incubated with anti-PNAG antibodies diluted 1:10.000 for 2 h, and these specific antibodies were detected with peroxidase-bound immunoglobulin G (goat anti-rabbit) antibodies (Jackson ImmunoResearch Laboratories, Inc., PA) diluted 1:10.000 by Western blotting system (Amersham ECL).

#### 2.4.4 Microtiter plate biofilm detachment assay

Biofilms were grown in sterile, 96-well, polystyrene microtiter plates (Iwaki, Asahi Glass Co. LDT, Japan) as described above. Biofilm detachment assays were carried out essentially as described in Kaplan et al. (2004). Biofilms were rinsed with water and treated with 200 μl of dispersin B (20 μg/ml in Tris or H_2_O), sodium metaperiodate (10 mM in 50 mM sodium acetate buffer at pH 4.5 or H_2_O, Sigma-Aldrich Chemie GmbH P.O., Steinheim, Alemania) or proteinase K (100 µg/ml in 20 mM Tris at pH 7.5–100 mM NaCl or H_2_O).

Control wells were treated with 200 μl of the appropriate buffer alone. After 30 min incubation at 30°C for treatment with dispersin B or 2 h at 37°C for treatment with sodium metaperiodate or proteinase K, biofilms were rinsed with water, stained, and quantified as described above. The biofilm detachment assays were performed in duplicate wells. All assays were performed at least twice, with similar results. Biofilm biomass was visualized, and quantities were obtained by quantitative biofilm assay as previously described and photographed (Supplementary Material, **Supplementary Figure S1A**).

### 2.5 Microbial adhesion studies

#### 2.5.1 Cell surface hydrophobicity

The CSH was measure by the Microbial Adhesion to Hydrocarbons (MATH) method (Reid et al., 1992), with some modifications. The bacteria were grown in TSB at 37°C to stationary phase (18–24 h of incubation). Then, the bacteria were washed three times with buffered saline (PBS) at pH 7.2, 0.01 M by centrifugation at 3000 rpm (~ 1000g) for 5 min. The bacterial suspensions were adjusted to optical density (OD) between 0.4 and 0.6, measured at a wavelength of 600 nm (DOi). On glass tubes previously washed with chromic solution, 3 ml of bacteria suspension was used. Later, 150 μl of hydrocarbon, n-hexadecane (Sigma, St. Louis, USA), was added and was stirred intensively with a vortex (Heidolph, Reax 2000) for two periods of 30 s, with an interval of 5 s in between. After 10 min at room temperature, both phases were completely separated, and the final optical density (DOf) of the aqueous phase was determined using a spectrophotometer (Helios Epsilon model, Thermo Spectronic, Waltham, MA, USA) at a wavelength (λ) of 600 nm.

In the case of *S. epidermidis* biofilm-producing strains, before the successive washings by centrifugation, the tubes were scraped off in order to obtain the highest number of cells that adhered to the walls. After washing, the suspension was homogenized by sonication for 5 min.

For measurement of hydrophobicity, the percentage of microorganisms that have moved from the aqueous phase in which they were suspended until the hydrocarbon phase after agitation (**Supplementary Material**, **Supplementary Figure S1B**) was calculated according to the following formula:


$$\%\text{CSH}=\frac{\text{ODi}-\text{ODf}}{\text{ODi}}\;\times \;100$$

The strains were classified according to their values of CSH, within the following ranges: 0–30% CSH, low hydrophobicity or hydrophilic; 30–60% CSH, moderate hydrophobicity; and 60–100% CSH, high hydrophobicity or hydrophobic. The experiments were performed at least three times, in different days. PBS suspensions were used as negative control.

#### 2.5.2 Adhesion to polystyrene

The strains were inoculated on blood agar (OXOID LTD., Basingstoke, Hampshire, UK) at 37°C for 24 h and were then grown in TSB for obtaining cultures in exponential state. The cultures were adjusted to a turbidity equivalent to 0.5 McFarland and diluted 1/100 to obtain an inoculum of approximately 10^6^ ufc/ml.

The adhesion to polystyrene was studied by spectrophotometry. For these experiments, the standard strains of *S. epidermidis* biofilm-producing (ATCC 35984 and ATCC 35983) and non-producing (ATCC 12228 and HAM 892) were selected.

Sterile, 96-well, flat-bottom polystyrene microtiter plates, both transparent and opaque (Greiner bio-one, Frickenhausen, Germany), were used. The bacterial inoculum was prepared from a culture at exponential phase in TSB and was centrifuged at 3.000 rpm for 5 min and resuspended in TSB twice. To homogenize the suspension, it was sonicated for 5 min before adjusted to an OD of 0.4 to 0.6 at a wavelength of 600 nm by spectrophotometer. Approximately 100 μl of the bacterial suspension was incubated for 1 h at 37°C. The time-elapsed bacteria were removed and washed twice with PBS. The number of bacteria attached to the bottom of the wells was measured by two different protocols. The OD was measured for each well at a wavelength of 492 nm using a microplate reader (ELx800, Bio-Tek Instruments, Inc, Winooski, VT, USA).

For adhesion to fibronectin-coated polystyrene (FN; Sigma-Aldrich Chemie GmbH P.O., Steinheim, Alemania), the first step was to adhere fibronectin to the surface of the wells of sterile, 96-well, flat-bottomed polystyrene microtiter plates, both transparent and opaque (Greiner bio-one, Alemania). 100 μl of fibronectin solution at a concentration of 50 μg/ml in sodium carbonate buffer was added to the wells and allowed to incubate for 18 h at 4°C according to existing methodology (Hussain et al., 2001). After this time, the content of the wells was aspirated, and a first wash was performed with 3% BSA in TBS to remove the fibronectin not bound to the surface and to block unspecific sites. Subsequently, a second wash with TBS was performed. Non-specific interaction has been defined as that associated with polystyrene binding (TBS) and specific interaction as that mediated by fibronectin (FN+BSA+TBS).

#### 2.5.3 Scanning electron microscopy

The selected bacterial strain was incubated at 37°C for 24 h with TSB on sterile glass coverslip circles placed inside 24-well microtiter plates (BioLite 24-Well Multidish, Thermo Fisher Scientific, Rochester, NY. USA). After 24 h of incubation, the coverslips were carefully washed twice with sterile PBS to remove non-adherent bacteria.

The formed biofilms were fixed at room temperature with 3% vol/vol glutaraldehyde (Panreac Química SAU, Barcelona, Spain) for approximately 15 h. Then, samples were dehydrated in a graded ethanol series (30, 50, 70, 90, and 100% vol/vol), with each step lasting 1 h. After, the samples were dried in a vacuum chamber, coated with a thin layer of gold (≤ 5 nm) using an EMITECH K575K (Quorum Technologies Ltd., West Sussex, UK) sputter coater, and finally, the image was captured with a scanning electron microscope (HITACHI S-4800, Hitachi High-Technologie, Tokyo, Japan).

### 2.6 Statistical analysis

All assays were performed in duplicate and independently repeated at least three times using separate bacterial cultures to ensure reproducibility. Results were expressed as the mean ± standard deviation (SD) when applicable. The relationship between bacterial adhesion to various surfaces and biofilm formation/cell surface hydrophobicity was evaluated by linear regression analysis using Microsoft Excel (Microsoft Office 365). Associations between hydrophobicity, biofilm-associated genes, and *mecA* presence *vs.* antimicrobial resistance were assessed using Spearman’s correlation (ρ) and Mann-Whitney U test. Data analysis was performed using SPSS software for Windows, version 24 (IBM Corp., Armonk, NY, USA), and a p-value of <0.05 was considered statistically significant.

## 3 Results

### 3.1 Genetic characterization

**Table 1** shows genetic characterization of the strains studied. The presence of the *icaADBC* operon genes, including the *ica*R regulatory gene, was demonstrated by amplification of the corresponding fragments. They were present in 44% of the clinical isolates (11/25). The *aap* and *bhp* genes involved in the formation of some biofilms were present in 48% (12/25) and 24% (4/25), respectively, and the methicillin resistance gene (*mecA*) in 52% (13/25). The genes for autolysin (a*tlE*), fibronectin-binding protein (e*mbp*), fibrinogen-binding protein (f*be*), and ATP-dependent protease (c*lp*P) were found in 100% of the strains. On the other hand, the insertion element *IS256* appeared in 40% (10/25) and *IS*257 was found in all strains. The amplicons were of the expected size, and no *IS256* insertions were detected. And as seen in **Table 1**, there was a higher correlation between the presence of the *icaADBC* operon and that of the *aap*, *bhp*, *IS256*, and *mecA* genes.

**Table 1:** Presence of *icaADBC*, *aap*, *bhp*, *atlE*, *fbe*, *embp*, *clpP*, *mecA*, *IS256*, *IS257*, and biofilm production in trypticase soy broth (TSB), measured by spectrophotometric test (AAG), production of “slime”, and Congo red agar (CRA) in *S. epidermidis* strains.

*S. epidermidis* strains		PCR fragments (bp)										*Biofilm* production		
		*ica ADBC*	*aap*	*bhp*	*atlE*	*fbe*	*embp*	*clpP*	*mecA*	*IS256*	*IS257*	G AA ^a^	Slime	CRA
*icaADBC*-negative strains (n=15)	ATCC 12228	–			+	+		+			+	0,32 ± 0.16	NP	NP
	54	–		+	+	+	+	+	+	+	+	0,42 ± 0.18	NP	NP
	73	–	+	+	+	+	+	+			+	0,27 ± 0.22	NP	NP
	1118	–			+	+	+	+			+	0,24 ± 0.16	NP	NP
	1162	–			+	+	+	+			+	0,29 ± 0.21	P	P
	1758	–			+	+	+	+			+	0,77 ± 0.14	P	NP
	1769	–			+	+	+	+	+		+	0,16 ± 0.10	NP	NP
	2739	–	+		+	+	+	+	+		+	0,25 ± 0.22	NP	NP
	2862	–			+	+	+	+	+		+	0,17 ± 0.13	NP	NP
	4054	–	+		+	+	+	+			+	0,35 ± 0.22	NP	NP
	4966	–			+	+	+	+			+	0,33 ± 0.05	NP	NP
	6560	–			+	+	+	+			+	0,22 ± 0.27	NP	NP
	6733	–	+		+	+	+	+	+		+	0,24 ± 0.23	NP	NP
	6735	–			+	+	+	+			+	0,31 ± 0.17	NP	P
	11063	–	+		+	+	+	+			+	0,48 ± 0.06	NP	NP
Presence (%) and mean ± SD		0	33.3	13.3	100	100	93.3	100	33.3	6.7	100	0,32 ± 0,17		
*icaADBC*-positive strains (n=14)	ATCC 35983	+			+	+	+	+			+	0.40 ± 0.08	P	P
	ATCC 35984	+	+	+	+	+	+	+	+	+	+	1.16 ± 0.15	P	P
	HAM 892	+	+	+	+	+	+	+	+	+	+	0,43 ± 0.20	NP	NP
	53	+			+	+	+	+	+		+	1,08 ± 0.09	P	P
	1701	+		+	+	+	+	+	+	+	+	0,74 ± 0.36	P	P
	2526	+	+		+	+	+	+		+	+	1,20 ± 0.47	P	P
	2868	+	+		+	+	+	+		+	+	0,33 ± 0.15	NP	NP
	2893	+	+		+	+	+	+	+	+	+	1,33 ± 0.45	P	P
	4061	+	+		+	+	+	+	+	+	+	0,54 ± 0.39	P	P
	4914	+			+	+	+	+	+	+	+	1,63 ± 0.24	P	P
	4942	+	+		+	+	+	+	+	+	+	0,35 ± 0.16	P	P
	6572	+	+		+	+	+	+	+	+	+	1,04 ± 0.05	P	P
	9542	+	+		+	+	+	+	+	+	+	0,80 ± 0.19	P	P
	9584	+		+	+	+	+	+			+	1,72 ± 0.20	P	P
Presence (%) and mean ± SD		100	64.2	28.6	100	100	100	100	71.4	78.5	100	0,91 ± 0,23		
All strains (n=29)		48.2	44.8	17.2	100	100	96.5	100	51.7	41.4	100			

a Quantitative measure of the biofilm production: Measurement to 492 nm according to the method of Stepanovic et al. with some modifications. The values are the average ± SD of at least three independent experiments by duplicate. NP, non-biofilm-producing strain; P, biofilm-producing strain.

### 3.2 Antimicrobial susceptibility testing

Among the 25 clinical isolates of *S. epidermidis* analyzed in this study, 18 (72%) were methicillin resistant. Notably, four of these methicillin-resistant strains (1162, 4054, 73, and 2868) did not carry the *mecA* gene (1162, 4054, 73, and 2868). Conversely, none of the seven methicillin-susceptible clinical isolates harbored the *mecA* gene. Aminoglycoside susceptibility testing (tobramycin, gentamycin, amikacin) revealed that 64% of the isolates (16/25) were susceptible to tobramycin, 60% (15/25) to gentamycin, and 64% (16/25) to amikacin. Penicillin G resistance was widespread, with only one isolate (6735) retaining sensitivity. Fluoroquinolone testing demonstrated high levels of susceptibility: 88% (22/25) to ciprofloxacin and 84% (21/25) to levofloxacin. All strains were susceptible to daptomycin and nitrofurantoin. Susceptibility rates were also high for mupirocin, fuchsidic acid, and fosfomycin (96%, 24/25); rifampicin (88%, 22/25); and linezolid (84%, 21/25). Moderate susceptibility was observed for trimethoprim/sulfamethoxazole (64%, 16/25), while lower rates were noted for tetracycline, erythromycin, and amoxicillin–clavulanic acid (52%, 13/25). Clindamycin showed the lowest susceptibility rate (36%, 9/25). Vancomycin MIC exceeded 2 mg/L in four clinical isolates, indicating reduced susceptibility. All four were methicillin resistant: two (4914 and 53) carried the *mecA* gene, while the remaining two did not. Further AST results are provided in **Supplementary Table S2**.

Among the 25 clinical isolates of *S. epidermidis* analyzed, 17 (68%) were classified as multidrug resistant (MDR), defined as exhibiting resistance to at least one agent in three or more distinct antimicrobial classes. Notably, several methicillin-resistant isolates also exhibited resistance to linezolid and trimethoprim/sulfamethoxazole, fulfilling the MDR criteria. The detailed distribution of resistant classes per strain is provided in **Supplementary Table S2**.

**Figure 1:**
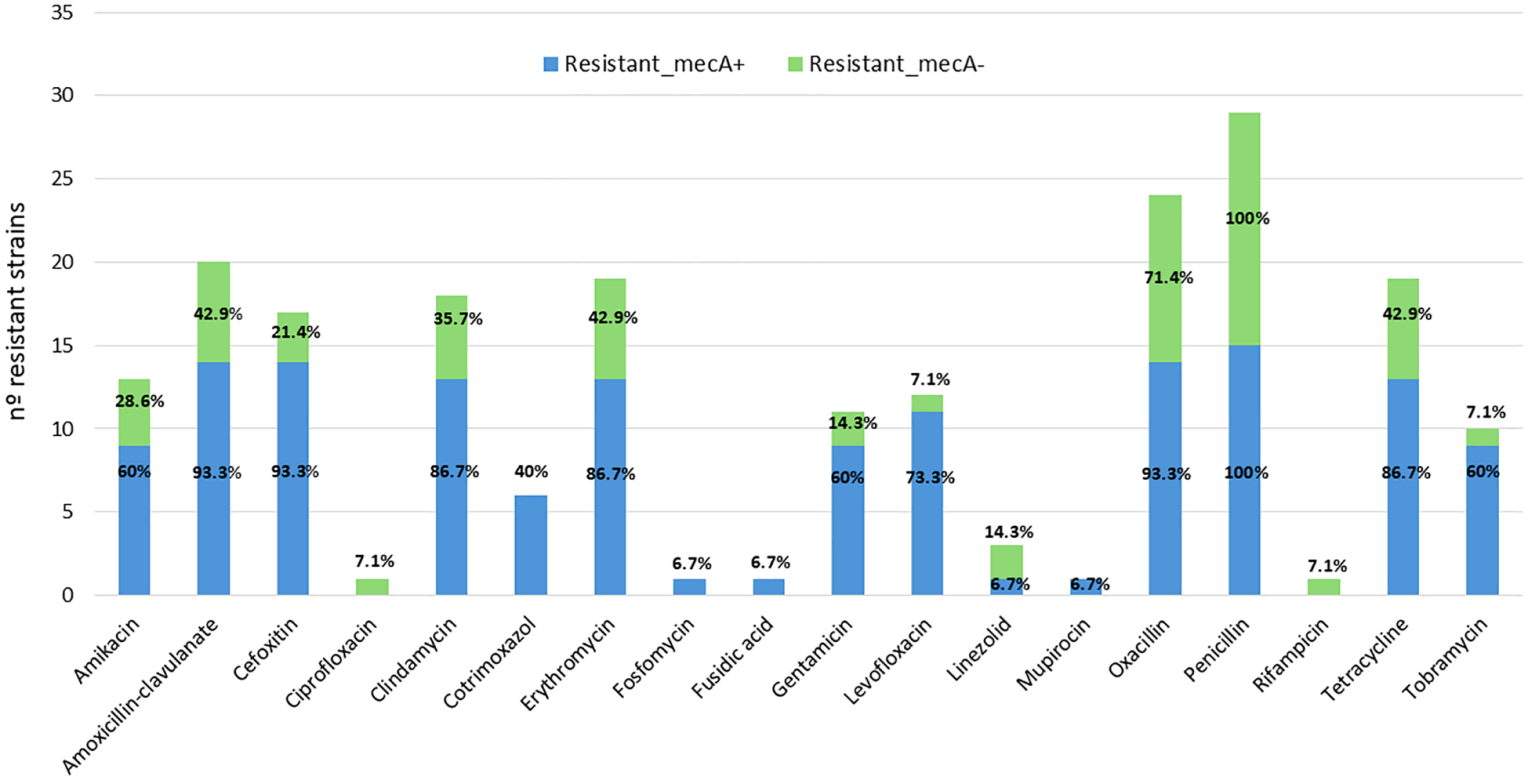
Distribution of antimicrobial resistance among *mecA*-positive (blue) and *mecA*-negative (green) *S. epidermidis* strains.

Attending to the presence or absence of *mecA*, *mecA*-positive strains show more overall resistance to multiple antibiotics (**Figure 1**). In particular, they show consistent resistance to cefoxitin, oxacillin, penicillin, clindamycin, tetracycline, amikacin, tobramycin, erythromycin, levofloxacin, amoxicillin–clavulanic acid, cotrimoxazole (various strains) (frequent), ciprofloxacin (some strains), fuchsidic acid, linezolid, mupirocin, and fosfomycin (in some strains). In contrast, *mecA*-negative strains show more variable and generally less extensive resistance patterns, although some are also resistant to key antibiotics (e.g., cefoxitin, amikacin, erythromycin, clindamycin, levofloxacin, etc.).

The analysis performed reveals a significant association between the presence of certain genes related to biofilm formation and the antimicrobial multidrug resistance profile in the *S. epidermidis* strains studied. The *IS256* and *mecA* genes showed the highest correlations with the number of antibiotics to which each strain was resistant, with Spearman correlation coefficients of ρ = 0.74 (p-value = 0.0001) and ρ = 0.73 (p = 0.0002), respectively. These results suggest that the presence of these genetic elements may be closely linked to the acquisition and maintenance of resistance determinants. The *icaADBC* gene, key in the synthesis of biofilm intercellular polysaccharide, also showed a significant positive correlation (ρ = 0.62, p = 0.0014), as did *aap*, which encodes a biofilm accumulation protein (ρ = 0.55, p = 0.0043). In contrast, the *bhp* gene, showed no significant association with antimicrobial resistance (ρ = 0.20, p = 0.31). These findings reinforce the hypothesis that resistance and persistence mechanisms are frequently linked in *S. epidermidis*, especially through elements such as *IS256* and the *ica* operon, underscoring their role in the pathogenicity of multidrug-resistant clinical strains.

When the comparative analysis is performed between biofilm-forming and non-biofilm-forming strains, a statistically significant difference was also observed in the number of antibiotics to which the strains are resistant (p-value = 0.009). This indicates that biofilm-forming strains tend to have a more pronounced multiresistance profile, supporting the role of biofilm not only in persistence but also in therapeutic resistance.

### 3.3 Quantitative and qualitative biofilm production

The qualitative and quantitative determinations of biofilm production are also shown in **Table 1**. Optimal correlation was found on the three assays. Most of the biofilm-producing strains showed colony morphology with rough texture and large biofilm formation in culture broth. Among the 11 *ica*-positive isolates, 10 were biofilm producers; while one of the strains possessed the operon, it was negative for biofilm production according to the two qualitative methods used in this study: slime production and Congo red agar. Among the 14 *ica*-negative strains, one formed biofilm by both methods (1162); however, two other strains had discrepancies. Strain 1758 was positive for the slime method while strain 6735 was positive for the CRA (**Table 1**).

Quantitative spectrophotometric determinations revealed that culture conditions influence biofilm production (see GAA standard deviation in **Table 1**), so we could not use it for the classification of biofilm-producing strains. In addition, the glacial acetic acid (GAA) method cannot discern whether it was a biofilm made up of a few bacteria producing a lot of slime or whether it was a lot of bacteria organized in a biofilm.

The qualitative Congo red agar method detects the production of various extracellular polysaccharides but not their adherence to surfaces or biofilm formation (Liberto et al., 2009). For this reason, the qualitative method of slime production was chosen to differentiate between biofilm-producing and non-biofilm-producing strains. Based on this criterion, we differentiated 12 biofilm-positive and 13 biofilm-negative strains.

Representative images corresponding to the phenotypic assays summarized in **Table 1** are shown in **Supplementary Table S2**. A clear phenotypic–genotypic discrepancy is observed in some strains: for example, *icaADBC*-negative strains 1162 and 1758 produce visible biofilm in the GAA and slime tests, whereas *icaADBC*-positive strain 2868 shows no biofilm production in any assay.

### 3.4 Presence of PIA/PNAG

**Figure 2:**
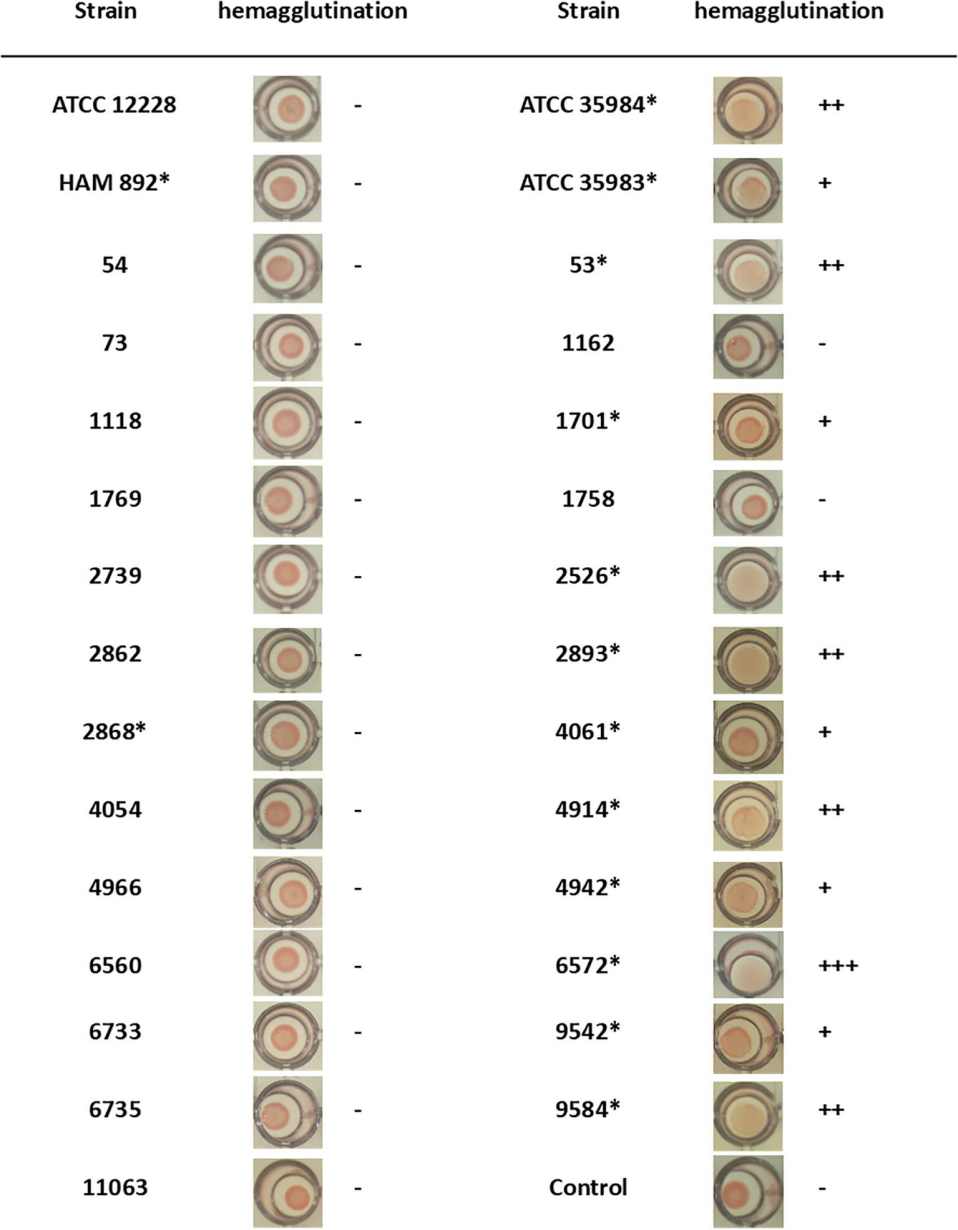
PIA/PNAG production of *S. epidermidis* strains by hemagglutination assay. Positive assay was determined when a diffuse deposit was visualized at the bottom of the well. Asterisk indicates *icaADBC*-positive strain.

A hemagglutination study was performed as a first approach to quantitatively test the presence of PIA/PNAG on the surface of 29 strains. It has been described that the PIA/PNAG molecule has a hemagglutinating effect (Rupp and Archer, 1992). Therefore, the demonstration of the hemagglutinating capacity of this bacterium is related to the presence of PIA/PNAG on the bacterial surface. Furthermore, in this way, it can be checked whether the data shown in the previous section are confirmed. **Figure 2** shows the images together with the interpretation of the assay for each of them. Of the 29 strains, 17 did not produce hemagglutination, while there were 12 strains that agglutinated red blood cells. It was found that of all strains with *icaADBC* operon, the strain 2868 provided a negative hemagglutination assay. This indicates that even though this strain has the genotype to form PIA/PNAG, this information cannot be translated on its phenotype. On the contrary, strains 1162 and 1758, which formed biofilm, do not present PIA/PNAG among their components as could be predicted, since they do not have the *icaADBC* operon (**Table 1**), which could be confirmed by this test by not producing hemagglutination (**Figure 2**).

For the qualitative study of PIA/PNAG presence by dot blot, 16 *S. epidermidis* strains with an unknown composition of their extracellular matrix were selected: 14 biofilm-producing strains (12 *icaADBC*-positive and 2 *icaADBC*-negative strains) and 2 non-biofilm-producing strains but containing the *icaADBC* operon (**Figure 2**).

**Figure 3:**
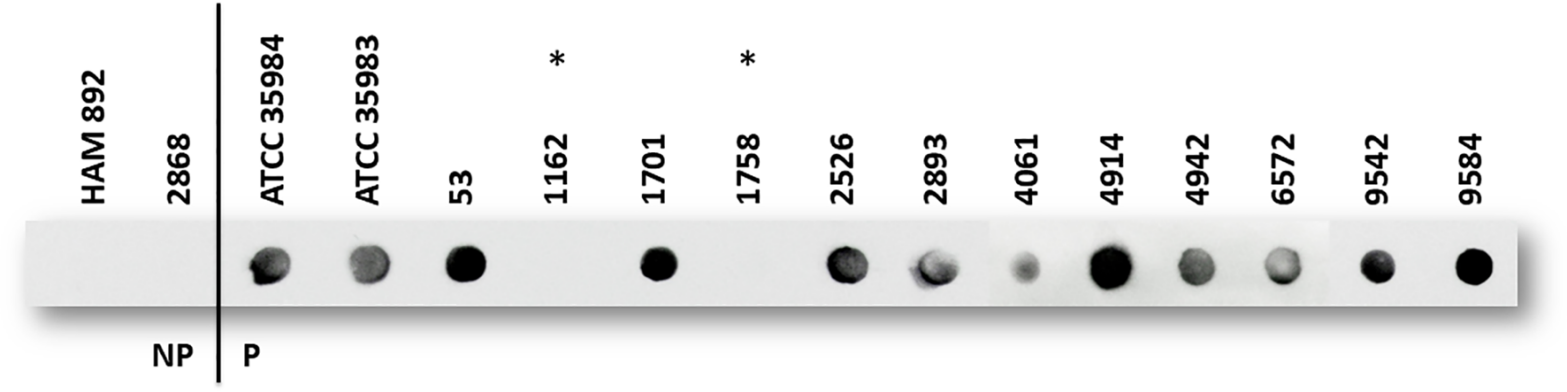
PIA/PNAG production of *S. epidermidis* strains by dot blot. A clear dot indicates the presence of the target, with darker or more intense dots generally suggesting higher concentrations. NP: non-biofilm-producing strain; P: biofilm-producing strain, according to “slime” assay. All strains are *Ica*+, except two strains with asterisk (*).

As shown in **Figure 3**, the *S. epidermidis* standard strains ATCC 35984 and ATCC 35983 and the clinical isolates of *S. epidermidis* 2526, 2893, 4942, 6572, and 9542 produced large amounts of PIA/PNAG. Even higher amounts were detected in *S. epidermidis* strains 53, 1701, 4914, and 9584. The biofilm of *S. epidermidis* 4061 produce a very small amount of PIA/PNAG. *S. epidermidis* 1162 and 1758 form an undetectable PIA/PNAG biofilm. Only *S. epidermidis* HAM 892 and 2868 do not produce PIA/PNAG despite the presence of the *icaADBC* operon in their genome.

### 3.5 Components of biofilms

Twelve biofilm-producing strains containing the *icaADBC* operon, and the *icaADBC*-negative strain 1758, which forms biofilm despite being *icaADBC*-negative, were chosen for this assay. Treatment was carried out with dispersin B (DspB), sodium metaperiodate (NaIO4), and proteinase K (PK) each dissolved in their corresponding buffers. DspB is an enzyme capable of hydrolyzing β-1,6-N-acetyl-D-glucosamines (PIA/PNAG). NaIO4 in slightly acidic medium selectively oxidizes C–C bonds, when both carbons possess hydroxyl or oxo groups, in short, oligosaccharide components, and PK hydrolyzes C-terminal peptide bonds of aromatic or hydrophobic amino acids. After the assays were performed, visualization of the biofilm and its disaggregation was carried out in 96-well microtiter plates by the glacial acetic acid (GAA) method (images are shown in the **Supplementary Material**, **Supplementary Table S3**).

**Figure 4:**
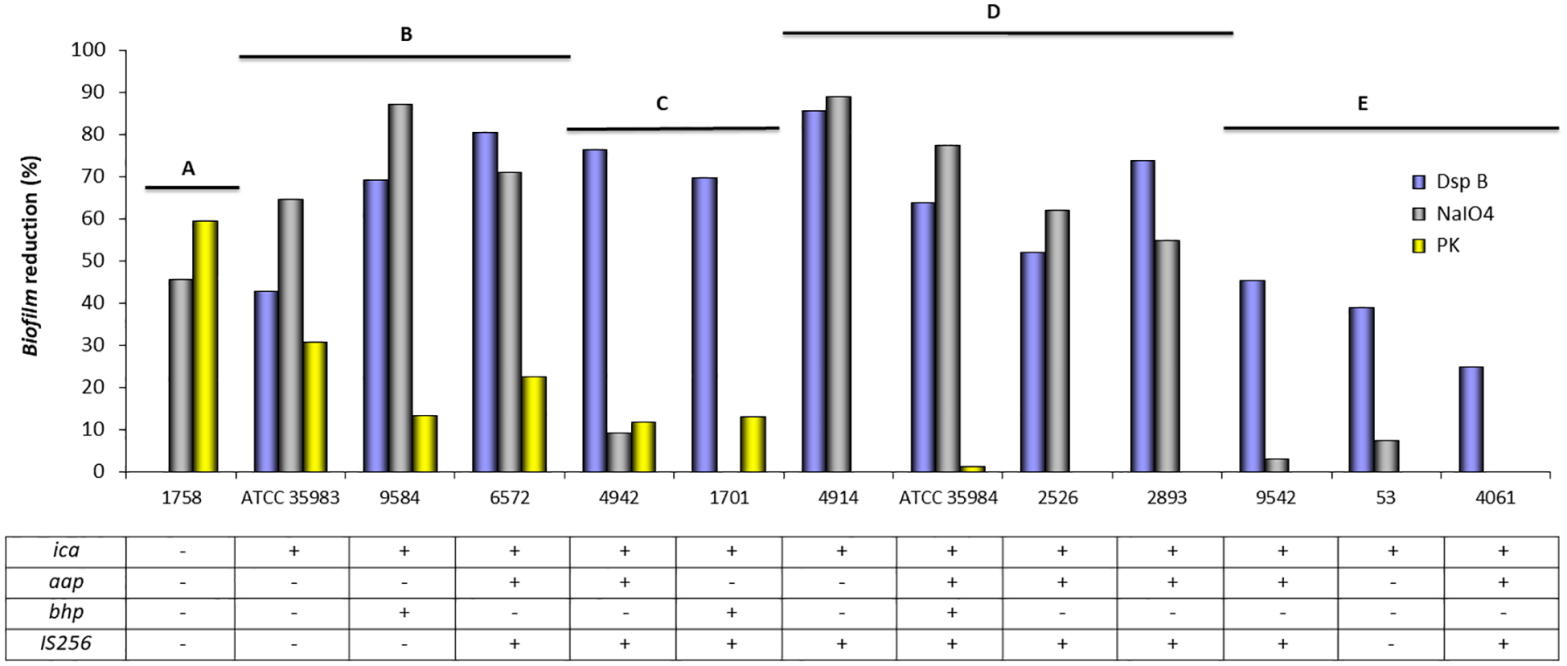
Mean of biofilm reduction (%) of *S. epidermidis* strains after treatment with dispersin B (DspB), sodium metaperiodate (NaIO_4_), and proteinase K (PK). The table indicates the presence (+) or absence (−) of genes related to biofilm formation.

The percentage of biofilm reduction by each treatment is described in **Figure 4**. It is observed that *S. epidermidis* ATCC 35984 (*ica+*, *aap+*, *bhp+*, *IS256+*) forms a mostly PIA/PNAG-dependent biofilm, which is not affected by proteinase K. In contrast, *S. epidermidis* ATCC 35983 (*ica+*), despite not containing *aap* or *bhp* in its genome, originates a biofilm composed of other possible proteins, because the biofilm is reduced to 27.4–30% after contact with proteinase K.

The biofilm of *S. epidermidis* strain 1758 (*ica−*, *aap−*, *bhp−*) was disintegrated by sodium metaperiodate (NaIO4) and proteinase K, confirming that it was composed of proteins and possibly other polysaccharide components, but not PIA/PNAG, as dot blot assay has also demonstrated.

The presence of PIA/PNAG in the biofilm composition of *S. epidermidis* strains 53 *(ica+*), 4061 (*ica+*, *aap+*, *IS256+*), and 9542 (*ica+*, *aap+*, *IS256+*) was confirmed by the ability of dispersin B to disintegrate the biofilm, which was however not affected by sodium metaperiodate (NaIO4) or proteinase K.

*S. epidermidis* 2526 (*ica+*, *aap+*, *IS256+*) and *S. epidermidis* 2893 (*ica+*, *aap+*, *IS256+*) formed a PIA/PNAG-dependent biofilm, since both dispersin B and sodium metaperiodate were able to disintegrate their biofilm. However, the biofilm was not affected by proteinase K action.

Dispersin B, sodium metaperiodate (NaIO4), and proteinase K can disintegrate the biofilm of *S. epidermidis* 6572 (*ica+*, *aap+*, *IS256+*) and 9584 (*ica+*, *bhp+*). Similarly, *S. epidermidis* 1701 (*ica+*, *bhp+*, *IS256+*) and 4942 (*ica+*, *aap+*, *IS256+*) formed a PIA/PNAG and protein-dependent biofilm, but sodium metaperiodate (NaIO4) was not able to disintegrate it.

In addition, 58.3% (7/12) of the biofilm-producing strains lacked the proteins susceptible to proteinase K disintegration.

**Figure 4** shows grouped strains according to biofilm quantification by spectrophotometric measurement. Different strains can be grouped according to their probable biofilm composition: A: unknown polysaccharides and proteins as possible components of the biofilm; B: PIA/PNAG, other polysaccharides and proteins as components of the biofilm; C: PIA/PNAG and proteins as possible components; D: PIA/PNAG and other polysaccharides as biofilm components; and E: PIA/PNAG as major component of the biofilm.

### 3.6 Cell surface hydrophobicity

**Figure 5:**
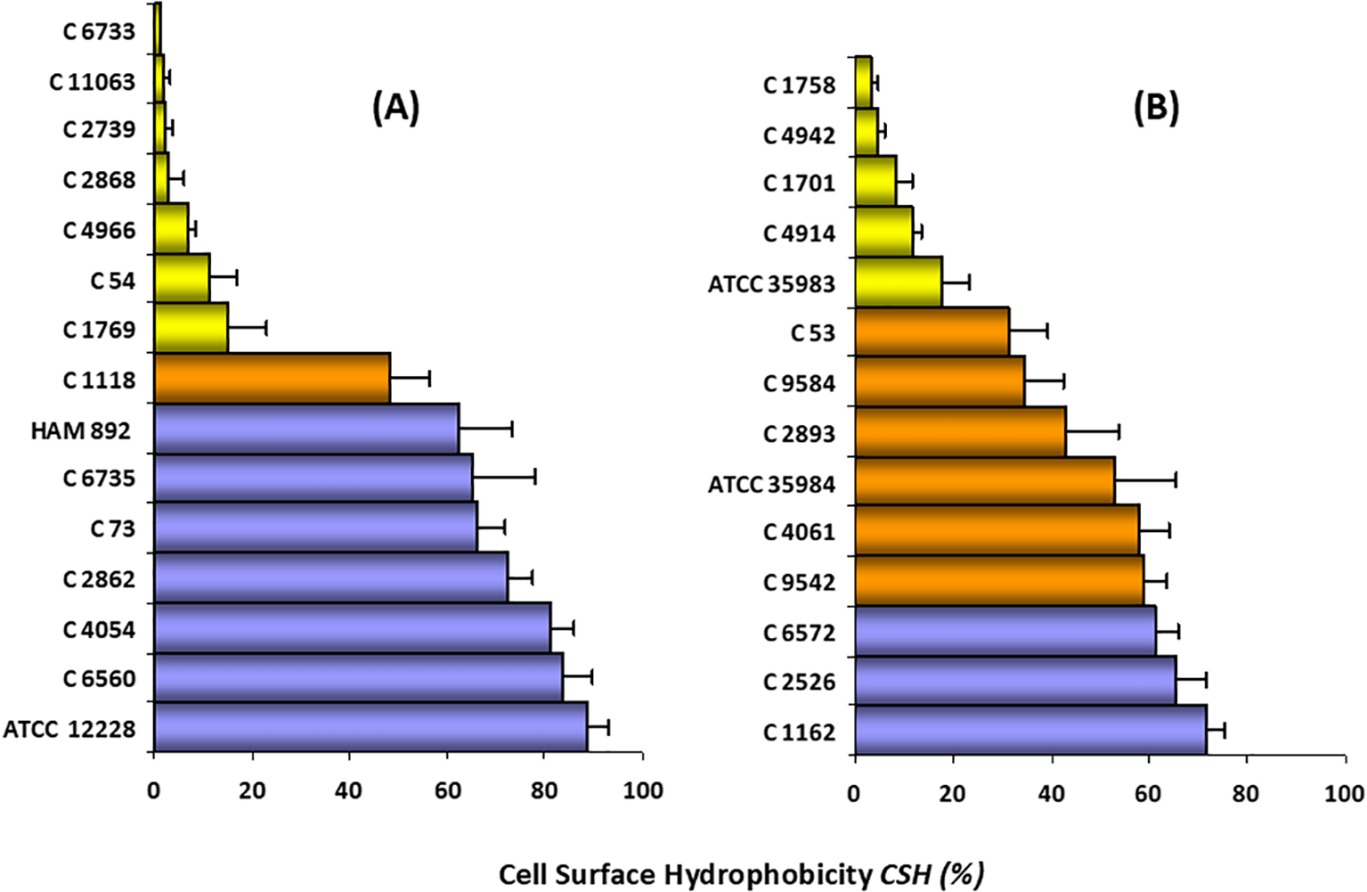
CSH classification of *S. epidermidis* strains. **(A)** non-biofilm-producing strains (n =1 5), **(B)** biofilm*-*producing strains (n = 14). Hydrophilic strains (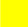); moderately hydrophobic strains (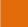); highly hydrophobic strains (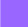). The bars represent the average ± standard deviation of at least three independent experiments. Ranges: 0–30% CSH, low hydrophobicity or hydrophilic; 30–60% CSH, moderate hydrophobicity; and 60–100% CSH, high hydrophobicity or hydrophobic.

**Figure 5** shows the CSH value by the MATH method for the 29 strains in the study (**Supplementary Material**, **Supplementary Table S4**). As can be seen, there is a wide range of values for CHS. 41.38% (12/29) of the strains were hydrophilic, 24.14% (7/29) showed moderate hydrophobicity, and 34.48% (10/29) were clearly hydrophobic.

For the non-biofilm-producing strains (n = 15) the CSH value ranged between 1 and 90%. 46.6% (7/15) of these strains were classified as hydrophilic, as they showed a CSH value of less than 30%. A similar proportion (7/15; 46%) was categorized as clearly hydrophobic (CSH > 60%). Only one strain, whose CSH was 48.23%, can be considered moderately hydrophobic.

Results of the biofilm-producing strains (n = 14) showed CSH values ranging from 3 to 71.5%. 35.7% (5/14) were classified as hydrophilic (range 3.3–17.8%), 42.9% (6/14) as moderately hydrophobic, and 21.4% of the strains (3/14) were considered as highly hydrophobic (CHS>60%).

As can be seen in **Figure 5** and **Supplementary Table S4**, there is a great heterogeneity in both groups of strains with respect to the CSH study. However, our results show that there is a high percentage of hydrophobic strains among the non-biofilm-producing strains (46.6% vs. 21.4%).

**Figure 6:**
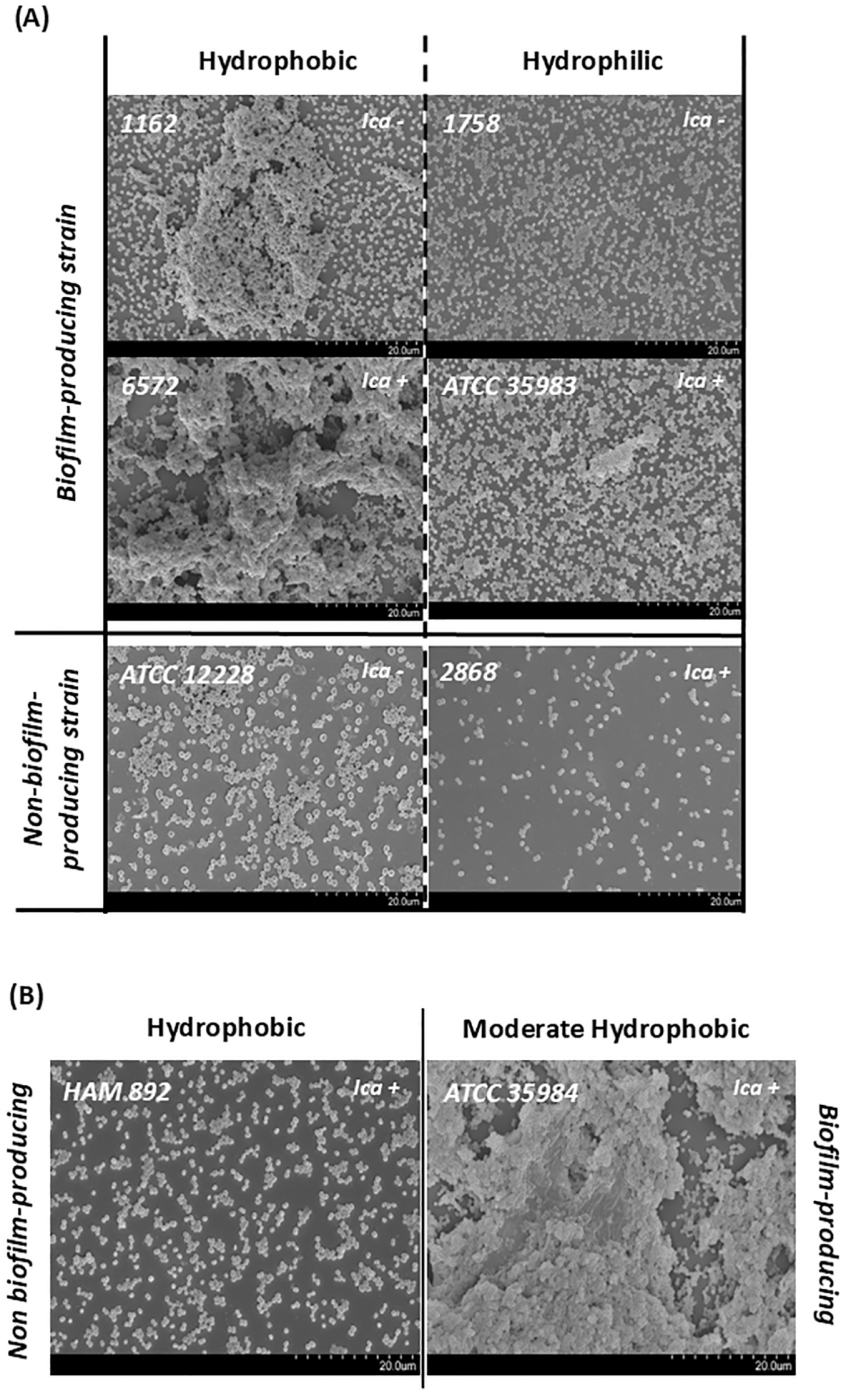
Scanning electron micrographs (2000×) of representative *S. epidermidis* strains illustrating surface adherence and biofilm architecture. Images are grouped according to their classification based on cell surface hydrophobicity (CSH). **(A)** Comparison of biofilm-producing and non-biofilm-producing strains with hydrophobic (left) and hydrophilic (right) surfaces. **(B)** Comparison of the highly biofilm-producing strain ATCC 35984 with moderate hydrophobicity and its mutant, HAM 892, a non-biofilm-producing strain with a highly hydrophobic surface.

Specifically, within the standard strains studied, the highest CSH was presented by the non-producing strain, followed by the mutant strain, the highly producing strain, and finally the moderately producing strain. Since strain HAM 892 is a mutant of the most productive strain, it can be said that the lack of exopolysaccharide substance on the surface of this non-producing strain provides higher CSH values than those of the wild-type strain. Both results suggest that CSH may be an important factor for adherence and colonization, especially in non-biofilm-producing strains. In fact, **Figure 6**, which illustrates the biofilm architecture of representative *S. epidermidis* strains using scanning electron microscopy, shows that the hydrophobic strains exhibit greater surface colonization compared to hydrophilic ones. Biofilm-producing strains with hydrophobic characteristics tended to form denser and more organized aggregates on the surface, whereas hydrophilic strains displayed looser and less compact structures.

SEM also confirmed key phenotypic–genotypic discrepancies shown in **Table 1**. Notably, strains 1162 and 1758, despite lacking the *icaADBC* operon, exhibited clear biofilm formation, easily observable with the slime test, supporting the existence of alternative aggregation mechanisms. Conversely, strain 2868, which is *icaADBC*-positive, did not form detectable biofilm structures under the tested conditions. In addition, strain HAM 892, a mutant derivative of the strong biofilm-producing reference strain ATCC 35984, showed impaired biofilm formation, in discrepancy with its genetic background, as expected. Interestingly, despite the absence of biofilm, strains ATCC 12228 and HAM 892 displayed robust surface adherence, likely facilitated by their high cell surface hydrophobicity. In contrast, strain 2868, which is hydrophilic, exhibited lower adhesion capacity, reinforcing the contribution of hydrophobicity to surface colonization.

### 3.7 Relationship between pathogenicity factors of *Staphylococcus epidermidis*

As discussed above (**Supplementary Table S4**), the CSH of the 29 strains ranged from 1 to 90%, and 14 strains produced biofilm (range 0.40–1.63). The possible relationship between the degree of CSH and the ability to form biofilm was analyzed, and no significant correlation was found between CSH and biofilm production, with a linear correlation coefficient of R2 = 0.00.

**Figure 7:**
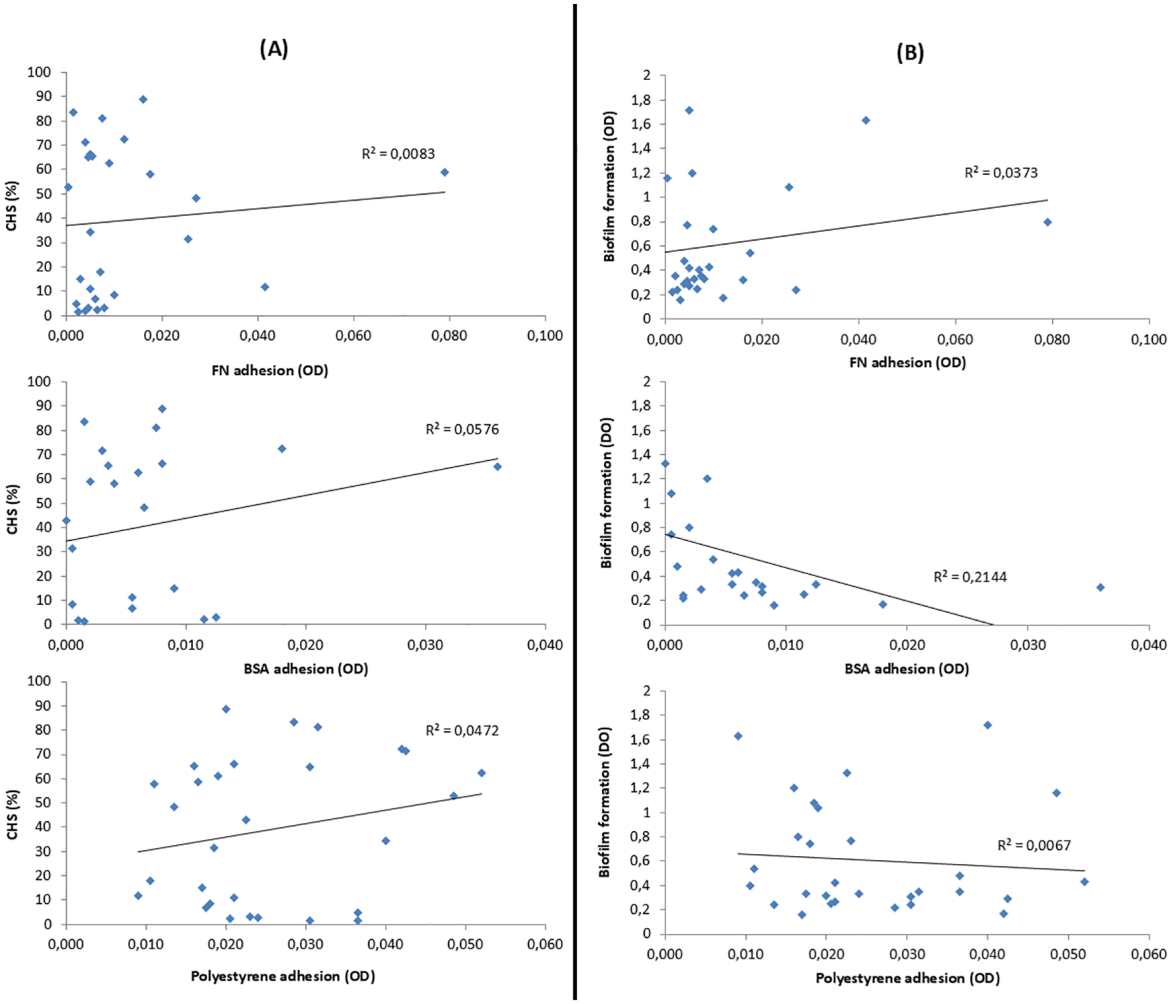
**(A)** Relation between the CSH of *S. epidermidis* and their adhesion to fibronectin (FN), bovine serum albumin (BSA), and polystyrene. **(B)** Relation between the biofilm formation of *S. epidermidis* and its adhesion to FN, BSA, and polystyrene. Correlation was evaluated using the coefficient of determination (R²); values closer to 1.0 indicate a stronger linear relationship, while values below 0.5 are considered to reflect weak or moderate correlation.

In order to prove if there was a relationship between CSH and adherence, both specific and non-specific ones, CSH data were related to FN, BSA, and polystyrene adherence data. These results are shown in **Figure 7A**. Similarly, adherence to FN, BSA, and polystyrene was also related to biofilm formation of the 29 *S. epidermidis* strains studied. These data are presented in **Figure 7B**.

Considering the CSH assay and focusing on the four standard strains, it can be deduced that a lower CSH leads to a greater specific interaction, as in the case of strain ATCC 35983, which, being the most hydrophilic, is the one with the highest FN adherence. On the contrary, highly biofilm-producing strains present a greater non-specific interaction, as in the case of strain ATCC 35984, which, being the most biofilm-producing of the four strains, is the one that presents the greatest adherence to polystyrene. Although, these considerations cannot be extrapolated to a larger population, since no optimal correlation between unspecific or specific adhesion with biofilm production or CSH of *S. epidermidis* strains was detected.

## 4 Discussion

The association between biofilm-related genes and virulence in *S. aureus* and *S. epidermidis* has been extensively studied (Kalantar-Neyestanaki et al., 2023; Monk et al., 2008; Otto, 2009). These studies frequently report that invasive strains of *S. epidermidis* more commonly harbored the *icaADBC* operon, while *ica*-negative strains are generally considered less virulent or non-virulent (Lin et al., 2021; Rohde et al., 2004). For instance, Rohde et al. observed a lower prevalence of *icaADBC* in commensal strains, whereas other genes involved in foreign body colonization, such as *AtlE*, *Fbe*, and *Aap*, did not show significant differences in distribution between invasive and commensal isolates.

In our study, we prioritized the biofilm-forming capacity of the isolates rather than their clinical or commensal origin. PCR analysis revealed that 44% of the strains carried the *icaADBC* operon. Notably, the presence of *aap*, *bhp*, *IS256*, and *mecA* was most frequent among *ica*-positive strains. These findings are consistent with previous reports, which indicate that the prevalence of the *ica* operon in clinical *S. epidermidis* isolates ranges from 20 to 60% (Cherifi et al., 2013; Harris et al., 2016; Martínez-Meléndez et al., 2016; Ahmed et al., 2019; Farajzadeh Sheikh et al., 2019).

The expression of *icaADBC* can be modulated by insertion sequences such as *I*S256, which has been shown to integrate into the operon and disrupt PIA production, converting biofilm-forming strains into PIA-negative variants (Kalantar-Neyestanaki et al., 2023; Kiem et al., 2004; Rohde et al., 2001).

In our dataset, *IS256* was detected in 78.5% of *ica*-positive strains and only in 6.7% of the *ica*-negative strains. However, we did not observe any PCR evidence of *IS256* insertion within the *ica* operon itself. This aligns with previous studies (Arciola et al., 2004; Petrelli et al., 2006; Bradford et al., 2006; Handke et al., 2004), which also failed to detect such insertions, suggesting that *IS256* may contribute to virulence through alternative mechanisms. Indeed, *IS256* is considered a marker of pathogenicity and genomic plasticity in *S. epidermidis* (Kalantar-Neyestanaki et al., 2023). There is increasing evidence that changes in gene expression during initial bacterial adhesion and intercellular adhesion (biofilm formation) offer promising avenues for new therapeutic interventions targeting *S. aureus* and *S. epidermidis* biofilm formation, presenting potentially advantageous alternatives to existing treatments (Paramanya et al., 2024).

Phenotypic assays revealed some inconsistencies between genotype and biofilm production. While 90.9% (10/11) of *ica*-positive clinical isolates formed biofilm, 14.2% (2/14) of *ica*-negative strains also exhibit biofilm-forming ability under our experimental conditions. Additionally, one *ica*-positive strain did not produce biofilm in the “slime” assay. These discrepancies have been previously reported (Van Kerckhoven et al., 2016) and highlight the complexity of biofilm regulation.

For example, Chokr et al. (2006) found that although the *ica* locus was highly prevalent among *S. epidermidis* strains isolated from implanted devices infections, the actual production of PIA and biofilm was lower. Their results, similar to ours, showed that 15% of strains formed biofilm without detectable PIA/PNAG (biofilm+, PIA−, *ica*+/−), while 8% did not form biofilm despite carrying the genes (biofilm−, PIA+, *ica*+). The existence of *ica*-independent mechanisms may help explain discrepancies between genotype and biofilm phenotype. Despite increasing research, the mechanisms that allow *S. epidermidis* to effectively transition from a planktonic lifestyle to a biofilm under the hostile environment generated by host-produced toxic chemicals have yet to be fully defined (Oliveira et al., 2023).

In our study, we observed considerable diversity in biofilm composition. *Ica*-negative biofilm-producing strains primarily formed protein and polysaccharide-based biofilms, while *ica*-positive strains produced PIA/PNAG-dependent biofilm. Both the hemagglutination and dot blot assays confirmed PIA/PNAG production in all *ica*-positive, biofilm-forming strains, with 33% (4/12) identified as overproducers (**Figure 3**). These results suggest that both protein components and PIA/PNAG contribute to biofilm stability.

When referring to CRA, among the 25 clinical strains of *S. epidermidis*, 48% (12) were CRA-positive and 83.4% (10 of 12) produced PIA/PNAG. None of the CRA-negative strains produced PIA/PNAG. These findings closely mirror those reported in a previous study involving 20 *S. epidermidis* strains, where 75% (15) were CRA-positive and 93.3% (14 out of 15) produced PIA/PNAG, while none of the CRA-negative strains did (Podbielska et al., 2010).

Sadovskaya et al. (2006) found that biofilm from orthopedic prostheses infections, regardless of PIA/PNAG production, consistently contained protein components and extracellular teichoic acids. They also noted varying degrees of polymer deacetylation, likely mediated by the *ica*B gen, and that biofilm susceptibility to enzymatic treatments correlated with matrix composition. Rohde et al. (2007) showed that biofilms from PIA/PNAG-positive strains were disrupted by dispersin B (DspB), but not by trypsin, while *ica*-negative strains were sensitive to trypsin but resistant to DspB—indicating protein-based adhesion as an alternative mechanism.

In our study, DspB effectively disrupted biofilms from standard strains *S. epidermidis* ATCC 35984 (RP62A), ATCC 35983, and most clinical isolates, confirming PIA/PNAG presence. However, DspB was ineffective against the *ica*-negative strain 1758. Sodium metaperiodate (NaIO_4_) showed similar effects but failed to disrupt biofilms in strains 53, 1701, 4061, 4942, and 9542 (**Supplementary Table S3**; **Figure 4**) despite high PIA/PNAG production in some (53 and 1701) as shown in **Figure 3**. This unexpected result has been previously addressed by Chaignon (Chaignon et al., 2007), who suggested that NaIO_4_ alone may be insufficient to degrade dense PIA/PNAG biofilms, as full depolymerization also requires mild acid hydrolysis. Biofilm composition may also vary with environmental conditions. For instance, *S. epidermidis* strains isolated from high-shear areas, such as the interior of catheters, tend to produce PIA-rich biofilms, in contrast to those found in low-shear environments (Schaeffer et al., 2016).

Proteinase K (PK), which cleaves peptide bonds, was ineffective against PIA/PNAG-rich biofilms produced by *S. epidermidis* strains ATCC 35984, 53, 2526, 2893, 4061, 4914, and 9545. However, it effectively disintegrated the biofilms formed by *ica*-negative strains 1758, ATCC 35983, 1701, 4942, 6572, and 9584 (**Supplementary Table S3**; **Figure 4**). Notably, in the case of strain 1758, PK was more effective than either DspB or NaIO_4_. Overall, PK only partially removed biofilm in fewer than 50% of tested strains, consistent with previous findings that proteinaceous biofilms are PK-sensitive, whereas PIA-based biofilms respond better to NaIO4 (Freitas et al., 2018).

Our results align with those of Qin et al. (2007), who found that NaIO_4_ disrupted 60% of ATCC 35984 biofilm but had no effect on *ica*-negative strains, while PK was ineffective against ATCC 35984 but disrupted biofilms from *ica*-negative strains. In our experiment, NaIO_4_ in H_2_O detached up to 77.4% of ATCC 35984 biofilm, whereas PK had no effect. In contrast, PK detached 59.5% of the biofilm from strain 1758. Neither *Aap* nor *Bhp* was detected in this strain, consistent with previous reports showing that not all biofilm-producing strains express the proteins (Hussain et al., 1997; Van Kerckhoven et al., 2016).

Other studies have described CoNS strains with biofilm composed mainly of teichoic acids and proteins, resistant to NaIO_4_, but partially degraded by PK (Kogan et al., 2006). Additionally, a higher prevalence of proteinaceous biofilms—especially in *S. epidermidis*—has been observed (Soumya et al., 2017). These authors proposed that NaIO4 sensitivity indicates PIA/PNAG presence, while resistance suggests its absence. However, in our study, five strains (4942, 1701, 9542, 53, and 4061) produced PIA/PNAG yet were resistant to NaIO_4_ (**Figure 4**). In two of these (4942 and 1701), PK was effective, but in the remaining three, neither treatment disrupted the biofilm. This highlights that some PIA/PNAG-producing strains can form biofilms resistant to conventional chemical degradation.

Based on our results, we conclude that *S. epidermidis* is capable of forming both polysaccharide-dependent and proteinaceous biofilms. The latter are often associated with the absence of the *ica* operon and rely on proteins such as *Bhp* and *Aap*, as well as eDNA, for structural integrity.

Infection development begins with bacterial adhesion to a surface—a complex, sequential process influenced by multiple factors, including cellular surface hydrophobicity (CSH). CSH is determined by various structural components such as membrane proteins, lipoproteins, phospholipids, and lipopolysaccharides. Therefore, it should not be evaluated solely from the thermodynamic perspective (Doyle, 2000).

Adhesion typically occurs in two phases: an initial phase governed by non-specific interactions (e.g., hydrophobicity and surface charge), followed by a specific phase involving bacterial adhesins, host receptors, and exopolysaccharide production. Therefore, selecting reliable methods to analyze the surface properties of staphylococci is essential. In this study we employed the MATH assay to characterize the CSH of 25 clinical isolates and 4 reference strains of *S. epidermidis*. In this method, a small volume of hydrocarbon is added to a bacterial suspension. Vigorous agitation creates hydrocarbon droplets to which bacterial cells may adhere, making it a convenient assay for assessing bacterial hydrophobicity (Doyle, 2000). We selected n-hexadecane, following the method proposed by Reid et al. with some modifications (Reid et al., 1992), due to its non-toxicity to microorganisms, ease of purification, low vapor pressure and freezing point below room temperature (Rosenberg and Doyle, 1990).

Analysis of the CSH revealed considerable heterogeneity among the *S. epidermidis* strains. Notably, hydrophobicity did not correlate with biofilm-forming capacity, consistent with findings from previous studies (Sánchez et al., 1989). Although CSH has been associated with biofilm formation and pathogenicity, our results showed a higher proportion of hydrophobic strains among the non-biofilm-producing biofilms group (46.6%) compared to the biofilm-producing group (21.4%).

Several authors have suggested that biofilms production in CoNS is associated with CSH (Schilcher and Horswill, 2020). Treatment with proteolytic enzymes such as trypsin has been shown to shift bacterial surfaces from hydrophobic to hydrophilic, while pepsin increases hydrophobicity. These changes in CSH following protease treatment indicate that hydrophobicity is determined, at least in part, by proteins or protein-associated molecules (Schiffer et al., 2019). Furthermore, studies on *S. aureus* have shown that biofilm-embedded cell exhibit increased surface hydrophobicity due to the presence of exopolysaccharide (Campanac et al., 2002). These authors also reported that repeated washings and resuspension can restore hydrophobicity, and that disruption of the biofilm’s three-dimensional structure reduces bacterial resistance to antiseptics such as quaternary ammonium compounds. This suggests that the extracellular matrix plays a key role in protecting biofilm-associated bacteria.

Our results indicate no clear correlation between hydrophobicity and biofilm formation capacity. This is consistent with the understanding that hydrophobic interactions are primarily involved in the initial stages of bacterial adhesion. Hydrophobicity is influenced by components such as teichoic acids, eDNA, and *AtlE*. In particular, *AtlE* has been shown to affect adhesion to plastic surfaces, likely due to the significant changes in surface hydrophobicity it induces. In contrast, biofilm formation typically occurs at later stages of infection (Otto, 2014). Among the reference strains analyzed, the highest CSH was observed in *S. epidermidis* ATCC 12228 (a non-biofilm producer), followed by HAM 892, ATCC 35984 (a strong biofilm producer), and ATCC 35983 (a moderate producer). Since HAM 892 is a mutant derived from a strong biofilm-producer strain, the absence of exopolysaccharide on its surface may explain its higher CSH compared to the wild-type strain. These findings, along with the higher proportion of hydrophobic strains among the non-biofilm-producing group, suggest that CSH may play a more critical role in initial adhesion and colonization, particularly in strains that do not produce biofilm. Therefore, targeting primary adhesion could be a promising strategy to prevent and facilitate more effective infection control.

During infection, staphylococci interact with both biotic and abiotic surfaces. While adhesion to host tissues involves specific interactions with matrix proteins such as fibronectin and fibrinogen, adhesion to abiotic materials—like polystyrene—relies more on physicochemical properties, including surface hydrophobicity and charge. Enzymes such as *AtlE* contribute to binding to abiotic surfaces such as polystyrene or glass (Schilcher and Horswill, 2020). *Aap* has been implicated in mediating these interactions (Conlon et al., 2014).

In our study, all strains adhered more strongly to uncoated polystyrene than to fibronectin-coated surfaces, suggesting that non-specific interactions dominate initial adhesion. Among the reference strains, ATCC 35983 (moderate biofilm producer) showed greater specific adhesion to fibronectin and was more hydrophilic, whereas ATCC 35984 (strong biofilm producer) adhered less to fibronectin and was more hydrophobic. However, these trends did not hold across the broader strain set (**Figure 7**), highlighting the complexity of adhesion mechanisms.

The lack of correlation between CSH, adhesion, and biofilm formation across strains may be explained by genetic variability. For instance, the absence of *aap* in strains 12228 and 35983 could account for their reduced adhesion to polystyrene, given *Aap*’s role in primary attachment. Additionally, the model-dependent nature of biofilm formation observed in clinical isolates supports the idea that environmental conditions significantly influence phenotypic expression.

Although no statistically significant association was observed between the hydrophobicity level of the strains and their antimicrobial resistance profile, our findings clearly show that biofilm-producing strains exhibit significantly higher levels of resistance compared to non-producers. This reinforces the clinical relevance of biofilm formation, not only as a mechanism of persistence but also as a factor closely linked to multidrug resistance in device-associated infections by *S. epidermidis*.

## 5 Conclusions

Infections associated with artificial devices implanted in the human body are common and represent a significant clinical challenge. *S. epidermidis* is a leading cause of these infections, primarily due to its capacity for adhesion and biofilm formation.

This study provides a comprehensive and functional insight into the pathogenicity factors of *S. epidermidis* in clinical strains, particularly in the context of device-associated infections. Unlike other studies focused on individual mechanisms, our results demonstrate that the virulence of *S. epidermidis* is driven by a complex combination of genetic and phenotypic factors.

The combined analysis of multiple genes associated with both initial adhesion and biofilm formation reveals significant relationship between the presence of the *icaADBC* operon and other determinants such as *aap*, *bhp*, *IS256*, and *mecA*, which may synergistically contribute to the establishment of persistent infections. The presence of *IS256*, *mecA*, and *icaADBC* genes is confirmed to correlate significantly with higher levels of antimicrobial resistance, reinforcing the hypothesis that biofilm-related genetic elements are closely linked to the persistence and multidrug-resistant nature of clinical strains of *S. epidermidis.*

Through the combined application of genetic biochemical and functional techniques, we have accurately correlated the genotype of the strains with their actual capacity to form biofilms. This approach has allowed us to identify strains that, despite carrying typical virulence genes, do not exhibit the corresponding phenotype under *in vitro* conditions, suggesting a complex regulation of gene expression, possibly mediated by mobile elements such as *IS256*. Biofilm-producing strains in the absence of the *icaADBC* operon have been characterized with extracellular matrices composed of proteins and other polysaccharides distinct from PIA/PNAG, confirming the existence of alternative cell aggregation mechanisms. This underscores the role of factors such as *Aa*p, *Bhp*, or *Embp* in the formation of *ica*-independent biofilms.

The results obtained from hydrophobicity assays, which are scarcely explored in previous literature, suggest that this surface property could be a pathogenicity factor itself. A high proportion of non-biofilm-producing strains exhibited marked hydrophobicity, suggesting that this characteristic could facilitate initial adhesion to abiotic surfaces or host tissues, even in the absence of an organized matrix.

Finally, this work highlights the utility of combining molecular and phenotypic tools to improve the discrimination between invasive and contaminant strains in clinical settings. The simultaneous characterization of virulence factors, antibiotic resistance, hydrophobicity, and biofilm-forming capacity could be the key to designing more effective preventive strategies and optimal treatments for *S. epidermidis*-associated infections.

## Data Availability

The raw data supporting the conclusions of this article will be made available by the authors, without undue reservation.
