# Supplementary material for: Biofilm formation and role of other pathogenic factors in the virulence of Staphylococcus epidermidis clinical isolates
Source: Front Cell Infect Microbiol. 2025 Aug 6;15:1630341. doi: 10.3389/fcimb.2025.1630341 (PMC12364849; doi:10.3389/fcimb.2025.1630341)
Supplement: Supplementary file 1 [file Table1.docx]

**Supplementary Materials**

**Figure S1.** Determination of pathogenicity factors. **A)** biofilm production by Semi-quantitative method (absorbance at 492nm) and qualitative methods (Slime production in tube and biofilm production in congo red agar). **B)** Cell surface hydrophobicity: Imagen of *S. epidermidis* hydrophilic strain, ATCC 35984 (1) and hydrophobic strain, ATCC 12228 (2).


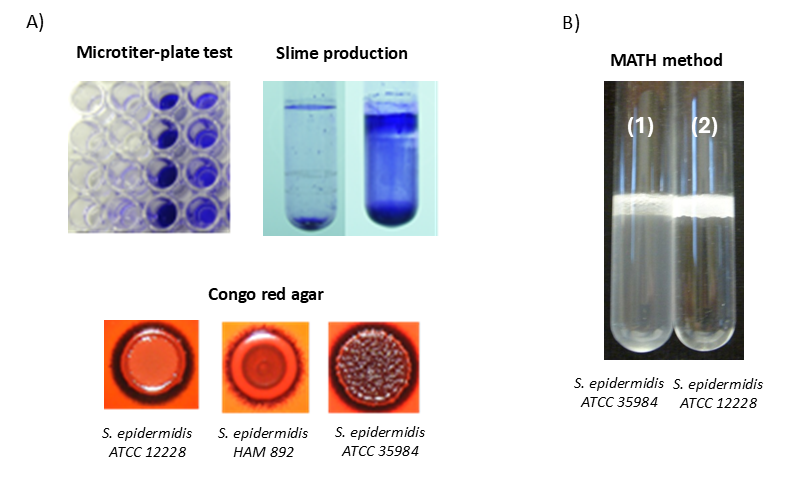


**Figure S2.** Phenotypic evaluation of biofilm formation in *S. epidermidis* clinical strains using three standard assays. Panel A shows strains lacking the icaADBC operon, while panel B includes icaADBC-positive strains. Each strain was tested using: (i) CRA (Congo Red Agar) plates, where black colonies indicate slime production; (ii) GAA (glacial acetic acid) assay, visualized first by crystal violet staining; and (iii) Slime test in glass tubes using Christensen’s method.


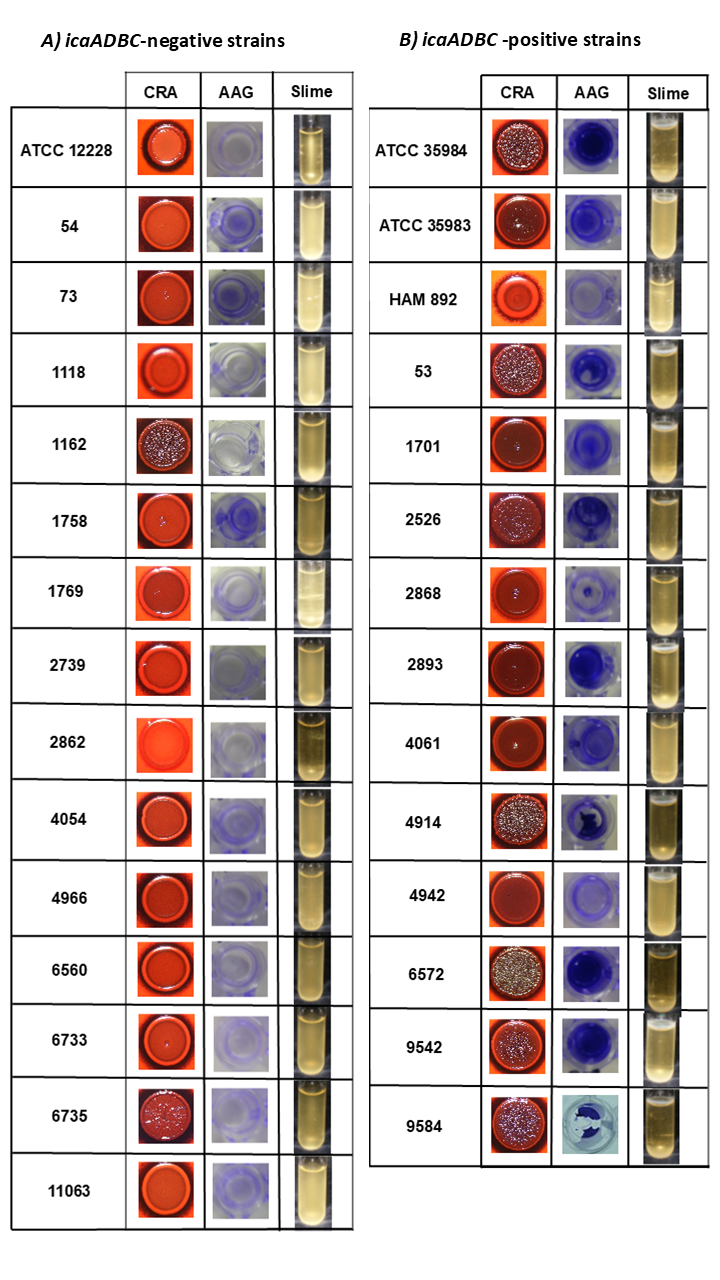


**TABLE S1.** Primers and conditions of the polymerase chain reaction (PCR), for genetic analysis of strains studied.

| **Gen^a^**  ***S. epidermidis*** | ***Primers*** | | **Reference** | **Study conditions** |
| --- | --- | --- | --- | --- |
|  | **Forward 5’-3’** | **Reverse 5’-3’** |  |  |
| ***icaA*** (221pb) | CTG TTT CAT GGA AAC TCC | TCG ATG CGA TTT GTT CAA ACA T | [[1](#_ENREF_1)] | 6min 94°C; 32 cycles [50s 94°C (denaturation), 1min 42°C (banding), 30s 72°C (elongation)], 5min 72°C. |
| ***icaD*** (197 pb) | ATGGTCAAGCCCAGACAGAG | CGTGTTTTCAACATTTAATGCAA | [[2](#_ENREF_2)] | 6min 94°C; 32 cycles [50s 94°C (denaturation), 1min 51°C (banding), 30s 72°C (elongation)], 1min 72°C. |
| ***icaB*** (328 pb) | TGG ATC AAA CGA TTT ATG ACA | ATG GGT AAG CAA GTG CGC | [[1](#_ENREF_1)] | 6min 94°C; 32 cycles [50s 94°C (denaturation), 1min 48°C (banding), 30s 72°C (elongation)], 5min 72°C. |
| ***icaC*** (99 pb) | GGC GTC GGA ATG ATG TTA AG | AAT TCC AGT TAG GCT GGT ATT G | [[1](#_ENREF_1)] | 6min 94°C; 32 cycles [50s 94°C (denaturation), 1min 51°C (banding), 30s 72°C (elongation)], 1min 72°C. |
| ***icaR*** (468 pb) | TAATCCCGAATTTTTGTGAA | AACGCAATAACCTTATTTTCC | [[3](#_ENREF_3)] | 6min 94°C; 32 cycles [50s 94°C (denaturation), 1min 48°C (banding), 30s 72°C (elongation)], 5min 72°C. |
| ***app*** (465pb) | AAA CGG TGG TAT CTT ACG TGA A | CAA TGT TGC ACC ATC TAA ATC AGC T | [[4](#_ENREF_4)] | 6min 94°C; 32 cycles [50s 94°C (denaturation), 1min 51°C (banding), 30s 72°C (elongation)], 2min 72°C. |
| ***bhp*** (1582pb) | ATG GTA TTA GCA AGC TCT CAG CTG G | AGG GTT TCC ATC TGG ATC CG | [[4](#_ENREF_4)] | 6min 94°C; 32 cycles [50s 94°C (denaturation), 1min 55°C (banding), 1.5min 72°C (elongation)], 5min 72°C. |
| ***atlE*** (681pb) | CAA CTG CTC AAC CGA GAA CA | TTT GTA GAT GTT GTG CCC CA | [[4](#_ENREF_4)] | 6min 94°C; 32 cycles [50s 94°C (denaturation), 1min 58°C (banding), 45s 72°C (elongation)], 2min 72°C. |
| ***fbe*** (272pb) | CTA CAA GTT CAG GTC AAG GAC AAG G | GCG TCG GCG TAT ATC CTT CAG | [[4](#_ENREF_4)] | 6min 94°C; 32 cycles [50s 94°C (denaturation), 1min 66°C (banding), 30s 72°C (elongation)], 2min 72°C. |
| ***embp*** (454pb) | AGC GGT ACA AAT GTC AAT | AGA AGT GCT CTAG CAT CAT CC | [[5](#_ENREF_5)] | 6min 94°C; 32 cycles [50s 94°C (denaturation), 1min 50°C (banding), 30s 72°C (elongation)], 2min 72°C. |
| ***clpP*** (127pb) | GGTATGGCAGCGTCTATGGGTT | ATTTCAGTTGCTTGTCCTTGTGC | This work | 6min 94°C; 32 cycles [50s 94°C (denaturation), 1min 66°C (banding), 30s 72°C (elongation)], 2min 72°C. |
| ***mecA*** (147pb) | GAA ATG ACT GAA CGT CCG AT | GCG ATC AAT GTT ACC GTA GT | [[4](#_ENREF_4)] | 6min 94°C; 32 cycles [50s 94°C (denaturation), 1min 47°C (banding), 30s 72°C (elongation)], 1min 72°C. |
| ***IS256*** (1102pb) | TGA AAA GCG AAG AGA TTC AAA GC | ATG TAG GTC CAT AAG AAC GGC | [[5](#_ENREF_5)] | 6min 94°C; 32 cycles [50s 94°C (denaturation), 1min 50°C (banding), 1min 72°C (elongation)], 5min 72°C. |
| ***IS257*** (560pb) | ACG TTC ATC ATT CAA CGG TC | AGT GTT CGC TTA ACT TGC TAG | [[4](#_ENREF_4)] | 6min 94°C; 32 cycles [50s 94°C (denaturation), 1min 47°C (banding), 30s 72°C (elongation)], 1min 72°C. |

^a^(PCR amplicons in base pairs). Primers designed from the sequence published in GenBank: accession numbers *icaADBC* (U43366), *aap* (AJ249487), *bhp* (AY028618), *mec A* (X52592), *IS256* (AF051917), *IS257* (U40381).

**TABLE S2.** Antimicrobial susceptibility testing of the 25 clinical isolates of *S. epidermidis.* A) MecA positive strains; B) MecA negative strains.

| **A)** | **MIC clinical isolates** | | | | | | | | | | | | | | | | | | | | | | | | | **MIC ATCC** | | | | | |
| --- | --- | --- | --- | --- | --- | --- | --- | --- | --- | --- | --- | --- | --- | --- | --- | --- | --- | --- | --- | --- | --- | --- | --- | --- | --- | --- | --- | --- | --- | --- | --- |
| **MecA positive strains** | **54** | | **1769** | | **2739** | | **2862** | | **6733** | | **53** | | **1701** | | **2893** | | **4061** | | **4914** | | **4942** | | **6572** | | **9542** | | | **HAM 892** | | | **35984** |
| Cefoxitin | >4 R | | >4 R | | >4 R | | <4 S | | >4 R | | >4 R | | >4 R | | >4 R | | >4 R | | >4 R | | >4 R | | >4 R | | >4 R | | | >4 R | | | >4 R |
| Gentamicin | >4 R | | <1 S | | <1 S | | <1 S | | <1 S | | <1S | | >4 R | | >4 R | | >4 R | | >4 R | | <1S | | >4 R | | >4 R | | | >4 R | | | >4 R |
| Amikacin | 16 R | | <8 S | | <8 S | | <8 S | | <8 S | | <8S | | >32 R | | >32 R | | >32 R | | >32 R | | <8S | | >32 R | | >32 R | | | >32 R | | | >32 R |
| Tobramycin | >2 R | | <1 S | | <1 S | | <1 S | | <1 S | | <1S | | >2 R | | >2 R | | >2 R | | >2 R | | <1S | | >2 R | | >2 R | | | >2 R | | | >2 R |
| Rifampicin | <0.5S | | >2 R | | <0.5 S | | <0.5 S | | <0.5 S | | <0.5S | | >2 R | | <0.5S | | <0.5S | | <0.5S | | <0.5S | | <0.5S | | <0.5S | | | <0.5S | | | <0.5S |
| Clindamycin | <0.25S | | <0.25 S | | 0.5 R | | >2 R | | <0.25 S | | >2 R | | >2 R | | 0.5R | | >2 R | | >2 R | | >2 R | | >2 R | | >2 R | | | >2 R | | | >2 R |
| Linezolid | 2 S | | <1 S | | 2 S | | 2 S | | 2S | | 2S | | 2S | | 4R | | <1S | | 2S | | <1S | | <1S | | 2S | | | 2S | | | 2S |
| Cotrimoxazol | >4/76 R | | >4/76 R | | <1/19 S | | <1/19S | | <1/19 S | | >4/76 R | | <1/19S | | >4/76 R | | <1/19S | | <1/19S | | <1/19S | | >4/76 R | | >4/76 R | | | >4/76 R | | | >4/76 R |
| Penicillin | >0.25 R | | >0.25 R | | >0.25 R | | >0.25 R | | >0.25 R | | >0.25 R | | >0.25 R | | >0.25 R | | >0.25 R | | >0.25 R | | >0.25 R | | >0.25 R | | >0.25 R | | | >0.25 R | | | >0.25 R |
| Vancomycin | 2 | | 1 S | | 2 S | | 2 S | | 2 S | | 4S | | 2S | | 2S | | 2S | | 4S | | 2S | | 1S | | 2S | | | 4S | | | 4S |
| Oxacillin | >2 R | | >2 R | | >2 R | | 2 R | | >2 R | | >2 R | | >2 R | | >2 R | | >2 R | | >2 R | | >2 R | | >2 R | | >2 R | | | >2 R | | | >2 R |
| Teicoplanin | 2 S | | 2 S | | 2 S | | 2 S | | 2 S | | 4S | | 2S | | 4S | | <1S | | 4S | | 2S | | <1S | | 4S | | | 4S | | | 4S |
| Daptomycin | <0.5 S | | <0.5 S | | 1 S | | 1 | | 1 S | | 1S | | 1S | | 1S | | <0.5S | | 1S | | <0.5S | | <0.5 | | 1S | | | 1S | | | 1S |
| Ciprofloxacin | <0.5 S | | <0.5 S | | <0.5 S | | <0.5 S | | <0.5 S | | <0.5S | | >2 R | | <0.5S | | >2 R | | <0.5S | | <0.5S | | <0.5S | | <0.5S | | | <0.5S | | | <0.5S |
| Tetracycline | >2 R | | <1 S | | >2 R | | >2 R | | >2 R | | >2 R | | >2 R | | >2 R | | <1S | | <1S | | <1S | | <1S | | 2R | | | >2 R | | | <1S |
| Erythromycin | <0.5 S | | >4 R | | <0.5 S | | >4 R | | <0.5 S | | >4 R | | >4 R | | <0.5S | | >4 R | | >4 R | | >4 R | | >4 R | | >4 R | | | >4 R | | | >4 R |
| Levofloxacin | <1 S | | <1 S | | <1 S | | <1 S | | <1 S | | <1S | | >4 R | | <1S | | >4 R | | 2R | | <1S | | <1S | | <1S | | | <1S | | | <1S |
| Fuchsidic acid | <2 S | | <2 S | | <2 S | | <2 S | | <2 S | | <2S | | >16 R | | <2S | | <2S | | <2S | | <2S | | <2S | | <2S | | | 16R | | | <2S |
| Fosfomycin | <32 S | | <32 S | | <32 S | | <32 S | | <32 S | | <32S | | <32S | | <32S | | <32S | | >64R | | <32S | | <32S | | <32S | | | <32S | | | <32S |
| Nitrofurantoin | <32 S | | <32 S | | <32 S | | <32 S | | <32 S | | <32S | | <32S | | <32S | | <32S | | <32S | | <32S | | <32S | | <32S | | | >64R | | | <32S |
| Mupirocin | <4 S | | <4 S | | <4 S | | <4 S | | <4 S | | <4S | | 255 R | | <4S | | <4S | | <4S | | <4S | | <4S | | <4S | | | <4S | | | <4S |
| Amoxicillin-clavulanate | >8/4 R | | <4/2 S | | <4/2 S | | 8/4 R | | <4/2 S | | 8/4R | | >8/4 R | | >8/4 R | | >8/4 R | | >8/4 R | | <4/2S | | >8/4 R | | >8/4 R | | | >8/4 R | | | >8/4 R |
| **B)** | | **MIC clinical isolates** | | | | | | | | | | | | | | | | | | | | | | | | | | | **MIC ATCC** | | |
| **MecA negative strains** | | 73 | | 1118 | | 1162 | | 1758 | | 4054 | | 4966 | | 6560 | | 6735 | | 11063 | | 2526 | | 2868 | | 9584 | | | 35983 | | | 12228 | |
| Cefoxitin | | >4 R | | <4S | | <4S | | <4S | | >4 R | | <4S | | <4S | | <4S | | <4S | | <4S | | >4 R | | <4S | | | <4S | | | >4 R | |
| Gentamicin | | <1S | | <1S | | <1S | | <1S | | >4 R | | <1S | | <1S | | <1S | | <1S | | >4 R | | >4 R | | <1S | | | >4 R | | | <1S | |
| Amikacin | | <8S | | <8S | | <8S | | <8S | | <8S | | <8S | | <8S | | <8S | | <8S | | >32 R | | >32 R | | <8S | | | >32 R | | | <8S | |
| Tobramycin | | <1S | | <1S | | <1S | | <1S | | <1S | | <1S | | <1S | | <1S | | <1S | | >2 R | | >2 R | | <1S | | | >2 R | | | <1S | |
| Rifampicin | | <0.5S | | <0.5S | | <0.5S | | >2 R | | <0.5S | | <0.5S | | <0.5S | | <0.5S | | <0.5S | | <0.5S | | <0.5S | | <0.5S | | | <0.5S | | | <0.5S | |
| Clindamycin | | 0.5R | | <0.25S | | 0.5R | | 0.5R | | >2 R | | 0.5R | | <0.25S | | 0.5R | | <0.25S | | <0.25S | | <0.25S | | 0.5R | | | >2 R | | | <0.25S | |
| Linezolid | | 2S | | 2S | | 2S | | 2S | | 4R | | 2S | | 2S | | 2S | | 2S | | 2S | | 2S | | 4R | | | 4R | | | 2S | |
| Cotrimoxazol | | >4/76 R | | <1/19S | | <1/19S | | <1/19S | | >4/76 R | | <1/19S | | <1/19S | | <1/19S | | <1/19S | | <1/19 | | >4/76 R | | <1/19S | | | <1/19S | | | <1/19S | |
| Penicillin | | >0.25 R | | >0.25 R | | >0.25 R | | >0.25 R | | >0.25 R | | >0.25 R | | >0.25 R | | <0.03S | | >0.25 R | | >0.25 R | | >0.25 R | | >0.25 R | | | >0.25 R | | | >0.25 R | |
| Vancomycin | | 2S | | 2S | | 4S | | 1S | | 2S | | 2S | | 2S | | 1S | | 2S | | 2S | | 4S | | 2S | | | 4S | | | 2S | |
| Oxacillin | | >2 R | | <0.25 | | >2 R | | <0.25 | | >2 R | | <0.25 | | <0.25 | | <0.25 | | <0.25 | | <0.25 | | >2 R | | >2 R | | | <0.25 | | | <0.25 | |
| Teicoplanin | | 2S | | <1S | | <1S | | <1S | | 2S | | 2S | | 4S | | <1S | | 2S | | 2S | | 4S | | 2S | | | 4S | | | 4S | |
| Daptomycin | | <0.5S | | 1S | | <0.5S | | 1S | | <0.5S | | <0.5S | | 1S | | <0.5S | | <0.5S | | 1S | | 1S | | 1S | | | 1S | | | 1S | |
| Ciprofloxacin | | >2 R | | <0.5S | | <0.5S | | <0.5S | | <0.5S | | <0.5S | | <0.5S | | <0.5S | | <0.5S | | <0.5S | | <0.5S | | <0.5S | | | 1S | | | <0.5S | |
| Tetracycline | | <1S | | <1S | | >2 R | | <1S | | <1S | | <1S | | >2 R | | <1S | | <1S | | >2 R | | >2 R | | <1S | | | >2 R | | | <1S | |
| Erythromycin | | >4 R | | <0.5S | | <0.5S | | <0.5S | | >4 R | | <0.5S | | >4 R | | <0.5S | | <0.5S | | <0.5S | | <0.5S | | <0.5S | | | >4 R | | | 2R | |
| Levofloxacin | | >4 R | | <1S | | <1S | | <1S | | <1S | | <1S | | <1S | | <1S | | <1S | | <1S | | <1S | | <1S | | | <1S | | | <1S | |
| Fuchsidic acid | | <2S | | <2S | | <2S | | <2S | | <2S | | <2S | | <2S | | <2S | | <2S | | <2S | | <2S | | <2S | | | <2S | | | <2S | |
| Fosfomycin | | <32S | | <32S | | <32S | | <32S | | <32S | | <32S | | <32S | | <32S | | <32S | | <32S | | <32S | | <32S | | | <32S | | | <32S | |
| Nitrofurantoin | | <32S | | <32S | | <32S | | <32S | | <32S | | <32S | | <32S | | <32S | | <32S | | <32S | | <32S | | <32S | | | <32S | | | <32S | |
| Mupirocin | | <4S | | <4S | | <4S | | <4S | | <4S | | <4S | | <4S | | <4S | | <4S | | <4S | | <4 | | <4S | | | <4S | | | <4S | |
| Amoxicillin-clavulanate | | >8/4 R | | <4/2S | | <4/2S | | <4/2S | | 8/4R | | <4/2S | | <4/2S | | <4/2S | | <4/2S | | <4/2S | | 8/4R | | <4/2S | | | <4/2S | | | <4/2S | |

**TABLE S3.** Biofilm degradation of biofilm-producing strains by dispersin B (Dsp B), sodium metaperiodate (NaIO_4_) and proteinase K (PK) on microtiter plates.


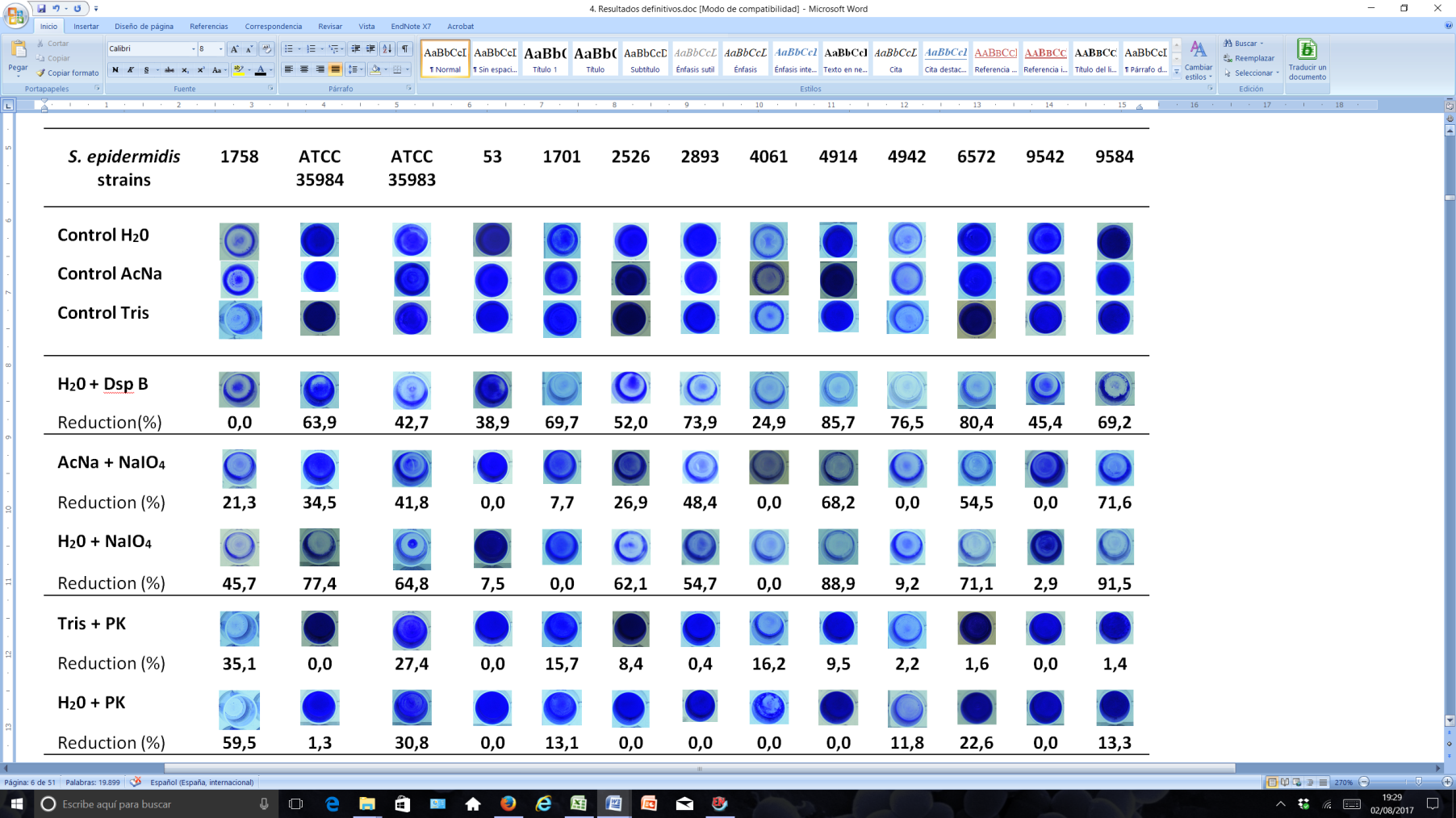


For this assay, biofilms were washed with water and then treated with dispersin B (DspB; 20 µg/mL in H_2_O) for 30 minutes, with sodium metaperiodate (NaIO_4_: in AcNa or H_2_O) for 2hours, and with proteinase K (PK; in Tris or H2O) for 2hours. The wells of the microplates were stained with crystal violet (CV) and then the dye was extracted with glacial acetic acid (GAA). The percentage of reduction was calculated with the values from the biofilm, readed with glacial acetic acid.

**TABLE S4.** Cell surface hydrophobicity and biofilm-forming values of *S. epidermidis* strains.

| ***S. epidermidis* strains** | | **CSH^a^ (%)** | ***Biofilm*^b^ (OD 492nm)** |
| --- | --- | --- | --- |
| **non-*biofilm*-producing (n=15)** | **ATCC 12228** | 88,86 ± 3,37 | 0,32 ± 0.16 |
|  | **HAM 892** | 62,51 ± 8,09 | 0,43 ± 0.20 |
|  | **54** | 11,10 ± 4,53 | 0,42 ± 0.18 |
|  | **73** | 66,17 ± 6,25 | 0,27 ± 0.22 |
|  | **1118** | 48,23 ± 2,65 | 0,24 ± 0.16 |
|  | **1769** | 15,05 ± 3,72 | 0,16 ± 0.10 |
|  | **2739** | 2,27 ± 2,16 | 0,25 ± 0.22 |
|  | **2862** | 72,33 ± 2,12 | 0,17 ± 0.13 |
|  | **2868** | 2,97 ± 0,80 | 0,33 ± 0.15 |
|  | **4054** | 81,23 ± 4,16 | 0,35 ± 0.22 |
|  | **4966** | 6,90 ± 2,38 | 0,33 ± 0.05 |
|  | **6560** | 83,40 ± 2,17 | 0,22 ± 0.27 |
|  | **6733** | 1,37 ± 1,21 | 0,24 ± 0.23 |
|  | **6735** | 65,07 ± 9,13 | 0,31 ± 0.17 |
|  | **11063** | 1,70 ± 2,21 | 0,48 ± 0.06 |
| ***biofilm*-producing (n=14)** | **ATCC 35983** | 17,84 ± 5,58 | 0.40 ± 0.08 |
|  | **ATCC 35984** | 52,75 ± 12,73 | 1.16 ± 0.15 |
|  | **53** | 31,46 ± 7,96 | 1,08 ± 0.09 |
|  | **1162** | 71,47 ± 3,97 | 0,29 ± 0.21 |
|  | **1701** | 8,40 ± 3,13 | 0,74 ± 0.36 |
|  | **1758** | 3,29 ± 1,44 | 0,77 ± 0.14 |
|  | **2526** | 65,47 ± 6,07 | 1,20 ± 0.47 |
|  | **2893** | 42,92 ± 10,72 | 1,33 ± 0.45 |
|  | **4061** | 58,04 ± 5,82 | 0,54 ± 0.39 |
|  | **4914** | 11,78 ± 1,60 | 1,63 ± 0.24 |
|  | **4942** | 4,78 ± 1,28 | 0,35 ± 0.16 |
|  | **6572** | 61,29 ± 4,70 | 1,04 ± 0.05 |
|  | **9542** | 58,78 ± 4,99 | 0,80 ± 0.19 |
|  | **9584** | 34,52 ± 7,94 | 1,72 ± 0.20 |

^a^The CSH was calculated using the MATH method. Values (%) of microbial adherence to n-hexadecane hydrocarbon represent the mean ± SD of at least 3 independent experiments as described by [[6](#_ENREF_6)]. ^b^Biofilm production values correspond to those obtained with the AAG method.

**References:**

[1] D. Mack, K. Bartscht, C. Fischer, H. Rohde, C. de Grahl, S. Dobinsky, M.A. Horstkotte, K. Kiel, J.K. Knobloch, Genetic and biochemical analysis of Staphylococcus epidermidis biofilm accumulation, Methods Enzymol 336 (2001) 215-39.

[2] K. Chaieb, K. Mahdouani, A. Bakhrouf, Detection of icaA and icaD loci by polymerase chain reaction and biofilm formation by Staphylococcus epidermidis isolated from dialysate and needles in a dialysis unit, J Hosp Infect 61(3) (2005) 225-30.

[3] C.R. Arciola, D. Campoccia, S. Gamberini, M.E. Donati, L. Montanaro, Presence of fibrinogen-binding adhesin gene in Staphylococcus epidermidis isolates from central venous catheters-associated and orthopaedic implant-associated infections, Biomaterials 25(19) (2004) 4825-9.

[4] H. Rohde, M. Kalitzky, N. Kroger, S. Scherpe, M.A. Horstkotte, J.K. Knobloch, A.R. Zander, D. Mack, Detection of virulence-associated genes not useful for discriminating between invasive and commensal Staphylococcus epidermidis strains from a bone marrow transplant unit, J Clin Microbiol 42(12) (2004) 5614-9.

[5] W. Ziebuhr, V. Krimmer, S. Rachid, I. Lossner, F. Gotz, J. Hacker, A novel mechanism of phase variation of virulence in Staphylococcus epidermidis: evidence for control of the polysaccharide intercellular adhesin synthesis by alternating insertion and excision of the insertion sequence element IS256, Mol Microbiol 32(2) (1999) 345-56.

[6] M. Rosenberg, D. Gutnick, E. Rosenberg, Adherence of bacteria to hydrocarbons - a simple method for measuring cell-surface hydrophobicity, Fems Microbiology Letters 9(1) (1980) 29-33.
